# Can Seaweed Be a Sustainable, One Health Resource in Livestock and Poultry Diets?

**DOI:** 10.3390/ani16193071

**Published:** 2026-09-29

**Authors:** Gerald C. Shurson

**Affiliations:** Department of Animal Science, College of Food, Agricultural and Natural Resource Sciences, University of Minnesota, St. Paul, MN 55108, USA; shurs001@umn.edu

**Keywords:** bioactive compounds, environmental impact, nutrients, One Health, poultry, ruminants, seaweed, sustainability, swine, toxic compounds

## Abstract

Seaweed is an abundant but underutilized resource in livestock and poultry feeds. The nutrient composition and digestibility are highly variable among and within seaweed species and geographic origin, and its ability to bioaccumulate several detrimental toxic substances in the marine environment can cause feed and food safety concerns. However, many seaweed species are rich sources of numerous bioactive compounds and phytochemicals that provide prebiotic, antioxidant, anti-inflammatory, antimicrobial, and antiviral properties as purified extracts that enhance animal productivity, health, and quality of animal-derived food products. Bromoform, which is found in high concentrations in red seaweed species, can be used as an effective dietary mitigation strategy for reducing enteric methane emissions from ruminants and subsequently reduce the environmental impact of meat and milk production. However, there are multiple tradeoffs between positive and negative environmental impacts, animal health benefits and risk factors, and improvements in quality of meat, milk, and eggs versus potential food safety concerns that are associated with the production, bioprocessing, and use of seaweed and bioactive compounds in animal diets that must be considered. This comprehensive review is intended to provide a synthesis of disparate threads of vast amounts of scientific information across multiple disciplines to help us answer the question “Can seaweed be a sustainable, One Health resource in livestock and poultry diets?”.

## Simple Summary

As global food animal production continues to increase to meet the increasing demand of meat, milk, and eggs from an increasing population of people, it must become less reliant on land and freshwater use, improve nutrient utilization efficiency, and reduce greenhouse gas emissions. Furthermore, disease pressures, elimination of antibiotic use for growth promotion, and oxidative stress in intensive livestock and poultry production systems have led to the need for identifying naturally produced anti-bacterial, anti-viral, antioxidant, anti-inflammatory, and immunomodulating compounds that can be used as feed additives in animal feed. Seaweed is an abundant but underutilized natural resource that can be utilized more extensively in livestock and poultry diets to overcome many of these environmental sustainability and One Health challenges of food animal production. However, there are many seaweed production, bioprocessing, and industry challenges that need to be overcome, and its beneficial detoxifying capabilities in the marine environment result in the bioaccumulation of several detrimental toxic substances with food and feed safety concerns. As a result, this comprehensive review is intended to provide a synthesis of disparate threads of vast amounts of scientific information across multiple disciplines to improve our understanding and help us evaluate the sustainability and One Health tradeoffs of seaweed use in animal feeds.

## 1. Introduction

Global food animal production has resulted in numerous ecosystem costs that include contributing 11 to 17% of total greenhouse gas emissions, utilizing 80% of land and 20% of freshwater resources to produce animal feed, disrupting nitrogen and phosphorus flows and contributing to freshwater eutrophication, directly and indirectly affecting biodiversity loss, and the development of antibiotic-resistant bacteria [1]. As food animal production continues to expand to feed an increasing human population, there is increasing pressure to identify and utilize alternative animal feed resources to reduce the burdens on land, freshwater and other natural resources while providing the necessary energy, nutrients, and nature-based pharmaceuticals to achieve optimal health, productivity, quality and safety of animal-derived food products that have low environmental footprints and achieve One Health goals. According to the World Health Organization, One Health is defined by the World Health Organization as “an integrated, unifying approach that aims to sustainably balance and optimize the health of people, animals, and ecosystems” [2]. This means that the health of humans, animals, plants, and all components of ecosystems are closely connected and interdependent.

Macroalgae (seaweed) is an abundant and underutilized natural and sustainable resource that has tremendous potential for overcoming many of these environmental sustainability and One Health challenges when used in animal feed. Seaweed production provides numerous environmental, economic, and social sustainability and One Health benefits [3,4,5,6,7]. Because seaweed grows in a marine environment, it does not compete with agricultural resources because no land, freshwater, or fertilizer is required. Furthermore, seaweed absorbs and sequesters significant amounts of carbon dioxide which substantially helps mitigate ocean acidification and global warming associated with climate change. Seaweed also purifies marine environments by removing nitrogen, phosphorus, heavy metals, and numerous toxic compounds. Several species of seaweed contain substantial amounts of bromoform, which has been shown to be very effective in reducing enteric methane emissions when fed to ruminants, but bromoform emissions from seaweed contribute to atmospheric halocarbon fluxes that may be associated with ozone depletion [7]. Many macroalgae species are rich sources of nutrients and bioactive compounds that can enhance animal health and productivity and may enhance meat and egg quality [8]. Producing more meat, milk and eggs using fewer feed and nutrient sources is essential for environmentally sustainable food animal production.

Increasing seaweed demand and use in animal feeds can result in several economic and societal benefits by creating a new economic sector based on marine resources that can provide alternative livelihoods for coastal communities to diversify income sources and avoid overfishing [3]. Seaweed has tremendous potential to increase income, food security, preserve marine biodiversity, and empower women and Indigenous peoples while decarbonizing other sectors as a regenerative and sustainable farming industry due to rapid growth, high yields, and low inputs [9]. Therefore, the production of seaweed enables increasing the market value of services provided by marine ecosystems.

Animal health and disease play a significant role in global food security and sustainability. Disease outbreaks decrease the supply of meat, milk and eggs resulting from high mortality rates and depopulation, cause chronic dietary nutrient use inefficiencies from subclinical infections, and lead to increased food prices and volatility, global trade restrictions, and loss of farmer livelihoods [10,11,12,13,14,15]. Although the environmental impacts of animal disease are poorly studied, high mortality causes nutrient losses and inefficiencies in production systems which increase carbon intensity and waste feed resources [16,17]. Furthermore, therapeutic and subtherapeutic use of antibiotics as growth promoters in livestock and poultry feed has contributed to the development of antimicrobial resistance, which has now become a major One Health crisis. As a result, many countries have banned or placed severe restrictions on antimicrobial use in livestock and poultry feeds, which has led to the need for identifying and exploring the use of numerous other naturally produced anti-bacterial, anti-viral, antioxidant, anti-inflammatory, and immunomodulating compounds that can be used as feed additives in animal feed. Many species of seaweed contain a wide variety of bioactive chemical compounds that can be extracted, purified, and used as feed additives in animal feeds to fill this gap [18,19,20,21].

However, we are stuck in a quandary of which comes first, the supply of seaweed or the demand for seaweed to build the industry and market for use in animal feeds? Should the seaweed industry expand production, bioprocessing capacity, and supply to meet the potential but uncertain demand from the animal feed industry, or should the livestock and poultry industry increase seaweed use in animal feeds to create more demand and incentivize the seaweed industry to expand to meet the need for increased supply? This dilemma has occurred because there is a major disconnect between seaweed producers and harvesters, seaweed bioprocessors, the animal feed industry and nutritionists, and the livestock and poultry producer sectors of the seaweed value chain. Stakeholders in each sector have a narrow view and limited awareness of opportunities and challenges in other sectors that affect the potential use of seaweed in animal feed. There are many factors that must be considered and limitations that must be overcome for seaweed, and the bioactive compounds it contains, to have a more prominent role in diets for food producing animals around the world. There have been no comprehensive reviews to synthesize all of this information together in one publication to help connect all segments of the seaweed supply chain, provide a “systems” overview to evaluate the multiple sustainability and One Health tradeoffs, and bridge the disconnect of understanding of the opportunities, challenges, benefits, and limitations of feeding seaweed to food producing animals. Therefore, the purpose of this review is to describe trends and challenges in global seaweed industry growth, production, and bioprocessing to meet emerging applications and demand in the feed, livestock, and poultry industry, and to summarize the nutritional and health benefits, feed and food safety concerns, and environmental impacts of using intact seaweed and extracts as a sustainable, One Health resource to produce meat, milk, and eggs. In this review, the term intact seaweed generally refers to any seaweed biomass that is harvested, dried, and processed to reduce particle size before adding to animal diets as a source of nutrients and bioactive compounds. Seaweed extracts are defined as purified substrates containing greater concentrations of specific bioactive compounds or mixtures than found in intact seaweed, which are obtained by various industrial processing and extraction methods, and are added to animal diets as feed additives.

## 2. Materials and Methods

To achieve the goals of writing this comprehensive, critical review, previously published systematic, scoping, meta-analysis, and critical reviews of peer-reviewed articles that used common search engines such as PubMed, Scopus, and ScienceDirect were relied upon to identify, cross-reference, and obtain original references on numerous topics involving seaweed and it use in animal feed. GoogleScholar, which is the largest multidisciplinary index, was used to search broadly and obtain peer-reviewed, systematic, scoping, meta-analysis, and critical review articles and original research studies related to seaweed between September 2025 to July 2026. More than 55 key words, terms, and variations on numerous topics involving seaweed production, bioprocessing, uses, and chemical composition; *in vivo* and *in vitro* studies involving dairy and beef cattle, sheep and goats, swine, broiler and layer chickens, ducks, geese, turkeys, and quails; and life cycle assessment (LCA) of environmental impacts of intact seaweed and extracts were used in the searches. Additional key words included One Health, agriculture, aquaculture, seaweed harvesting, seaweed farming, marine ecosystems, terrestrial ecosystems, environment, water, livestock, poultry, meat, milk, eggs, food, feed safety, food safety, nutrients, nutraceuticals, bioactive compounds, bromoform, toxicology, regulations, animal health, human health, economy, government policies, nutrient cycling, environmental contamination, analytical methods, heavy metals, antibiotic resistance, toxic environmental chemicals, and bioremediation. Documents from reputable international organizations such as World Bank, Food and Agriculture Organization of the United Nations, and others were used to accurately report industry statistics.

## 3. The Global Seaweed Industry

The global seaweed industry is a rapidly expanding, multi-billion-dollar sector of the global economy that has been mainly driven by a concentrated aquaculture industry in Asian countries dominated by China, Indonesia, and Republic of Korea, but there is increasing interest in expansion in other parts of the world. Between 2002 and 2022, global seaweed production increased from 11.9 to 36.3 million tonnes and the value of global exports quadrupled to $3.9 billion [22]. Most (97%) of the increased global production of seaweed has been due to cultivated seaweed farming rather than wild harvesting. Most of the seaweed produced is consumed locally in domestic markets, with only 0.75 million tonnes traded internationally in 2022 [22]. Due to current barriers to global trade which include high compliance costs and limited certification and processing capabilities, seaweed exports to other countries have been challenging, and require producers to develop processing capabilities and capacity for producing higher value extracts and derivatives. Therefore, capital investments in processing are essential for increasing value of seaweed products for export and domestic use.

Macroalgae provides many benefits to society including serving as a source of food nutrients, food hydrocolloids (agar, alginate, carrageenan), several chemical compounds used in cosmetics and pharmaceuticals, and have emerging applications in the production of bioplastics, biofuels, and animal feed. The World Bank [23] identified 10 global seaweed markets that have the potential to increase economic growth of the industry in the amount of $11.8 billion by 2030, which include short-term markets for biostimulants, animal feed, pet foods, and methane-reducing animal feed additives ($4.4 billion); intermediate-term markets for nutraceuticals, alternative proteins, bioplastics, and fabrics ($6 billion); and long-term markets for pharmaceuticals and construction applications ($1.4 billion).

In addition to its many useful applications, seaweed grows quickly, requires minimal inputs, removes pollutants from marine environments, sequesters carbon, and can provide food security, a source of income, and employment in local communities, which collectively contributes toward meeting at least nine of the 17 United Nations Sustainable Development Goals [9,23]. These sustainability benefits of seaweed farming will be the key drivers for industry growth as interest and demand for “green” products continues to increase [23].

However, there are numerous challenges that must be overcome before the seaweed industry can be utilized to its full potential. One of the major challenges across all markets is the lack of availability and consistency of seaweed supply and quality, which emphasizes the need for increasing primary production [23]. New and emerging markets must compete with established seaweed supply chains for use in human foods and processing facilities to produce hydrocolloids, which is challenging for obtaining competitive prices for other types of seaweed and uses [23]. Therefore, biorefineries need to be developed to extract several extracts and concentrated products from a single input of seaweed [23].

Another major challenge is incomplete and fragmented regulatory frameworks that have not kept pace with the rapid expansion of the seaweed industry, especially for new uses of seaweed derivatives [22]. For example, only 18 specific technical measures for seaweed have been identified, and focus mainly on food safety assessment, but measures of environmental standards and non-food uses of seaweed have not been adequately developed [22]. The lack of well-defined technical standards has created confusion and makes compliance with requirements in markets difficult to achieve for seaweed exporters. Furthermore, of the standards that do exist, they vary widely across countries resulting in national regulations that are not aligned with international standards. Because of these fragmented standards, the ability for small seaweed production businesses to export is difficult and costly due to the lack of trust in product quality and safety for buyers.

Recommendations [22] for further development of the global seaweed industry include:

- Develop science-based, harmonized standards to reduce these barriers to commerce while protecting consumers and ecosystems.
- Increase international cooperation, coordination, access to financing, and investment in research are needed, especially for small seaweed producers.
- Incentivize innovation in valorization, circularity, and integrate seaweed aquaculture into existing supply chains to increase economic value and resilience of local communities and countries.
- Develop better systems for classifying and tracking seaweed through supply chains to assist in aligning regulations, building capacity, developing more effective government policies, and supporting more inclusive growth across the seaweed industry.

There is tremendous potential for sustainable growth of the global seaweed industry that can help diversify and strengthen economies in an ever-changing world. However, without clear, harmonized standards, regulations, and increasing investment in infrastructure and research, the many advantages of seaweed production and use will not be completely realized.

## 4. Seaweed Production Systems

### 4.1. Locations and Major Seaweed Species Produced

Most (97%) of total global seaweed biomass production is derived from commercial seaweed farms with the remainder from wild harvest [24]. Currently, most (99%) of the global commercial seaweed production occurs in Asia (i.e., China, Indonesia, Japan, South Korea, Philippines; Table 1), with 96.5% of this total produced using well-established cultivation methods [24]. In contrast, the commercial seaweed industry in the U.S. and Europe is relatively undeveloped and mainly involves harvesting wild populations of macroalgae or production on small-scale aquaculture farms. There are more than 7934 green seaweed species classified under the *Chlorophyta* phylum, 7554 red seaweed species classified under the *Rhodophyta* phylum, and about 2220 brown seaweed species classified under the *Ochrophyta* (*Heterkontophyta* or *Phaeophyceae*) [25] found in the Earth’s oceans. However, there are only about 12 seaweed species that are commercially farmed globally, of which five seaweed genera accounted for 95% of global seaweed production with *Laminaria* and *Saccharina* accounting for 34.7%, *Kappaphycus* and *Eucheuma* representing 32.6%, with *Gracilaria*, *Porphyra*, and *Undaria* contributing 10.3%, 8.3%, and 7.2%, respectively [26].

### 4.2. Sustainability Challenges of Seaweed Production

Although wild harvest of seaweed represents a relatively minor proportion of the global seaweed supply, more than 800,000 tonnes of seaweed are harvested from natural seaweed beds annually with little concern regarding the sustainability of this practice [29]. As a result, over-harvesting to meet commercial demand has caused deterioration of seaweed beds and may affect the future ability to maintain markets for these specific supply chains [29]. Furthermore, seaweed that is washed to shorelines must be collected and dried relatively quickly to prevent decay and hydrogen sulfide emissions that can adversely affect human and animal health [30]. Although seaweed yields are greater for cultivated seaweed production systems than from wild harvest [31], intensive large-scale seaweed production systems are more vulnerable to acquiring diseases [32]. Many of the most commercially important species such as *Saccharina*, *Laminaria*, *Kappaphycus*, *Pyropia*, and *Eucheuma* are produced under increased disease pressures from endosymbionts and epibionts in intensive cultivation systems that can lead to significant economic losses [33]. Therefore, coupling either wild harvest or cultivated seaweed production systems with extensive and intensive livestock and poultry production systems has been risky for long-term resilience of supply chains and a major cause for the current disconnect between the seaweed, feed, and food animal production industries.

Seaweed production requires sunlight for photosynthesis, seawater or brackish water, dissolved carbon dioxide, macronutrients (nitrogen and phosphorus), and water movement to circulate nutrients. Climate change involving abiotic changes, extreme weather events, disease, and herbivory are affecting biomass yields, losses, and chemical composition of seaweed production [34]. The abiotic effects of ocean warming and acidification changes the seasonality of seaweed production which reduces biomass yields and alters the chemical composition. Increased frequency and severity of adverse weather events such as excessive heat, storms, and freshwater nutrient inputs cause losses and damage to seaweed farms. Warming ocean temperatures and decreased salinity increase environmental stress on seaweed which make it more vulnerable to diseases and the loss of herbivory habitat. To overcome these challenges, Veenhof et al. [34] proposed future proofing site selection to ensure environmental conditions are suitable for the specific species to be grown; utilizing improved genetic selection; engineering techniques and hybridization to improve stress tolerance, yields, and microbiome manipulation; and implementing restorative aquaculture practices.

### 4.3. Production and Harvest Methods

Seaweed production and harvest methods include natural collection of beach cast seaweed, harvest of wild stocks, extensive cultivation using nearshore or offshore mariculture systems, integrated multi-trophic aquaculture (IMTA), and intensive open land-based cultivation systems that use open ponds and tanks [35]. Amponsah et al. [35] summarized the distinguishing characteristics among these diverse production and harvesting systems.

Offshore seaweed farming occurs in open ocean areas beyond the coastal shoreline where water is deeper, richer in nutrients, and less affected by human activities and pollution [35]. Nearshore cultivation systems grow seaweed on artificial substrates located on the seabed and are suspended with floating rafts or long line methods using polypropylene rope or nylon lines located below the water surface of shallow lagoons and sandy sea bottoms [35]. However, despite the inexpensive design of these “off bottom” methods that enable manual maintenance, they are labor intensive [35]. Therefore, using rope-based methods in the open sea, closed bays, and estuaries are preferred [35].

Offshore or deep-sea seaweed cultivation systems are in very deep ocean water (>100 m) in less protected areas away from shorelines to minimize competition with fishing, tourism, oil and gas extraction, wind energy, and ocean transport industries for offshore space [35]. These systems use anchoring and floating technology that allow seaweed to grow under low light and high-pressure conditions, but they are exposed to high wind and wave conditions [35]. Implementation of these types of seaweed cultivation systems (i.e., vertical or horizontal long lines, floatation devices, cultivation rings, and integration with wind and shellfish farms) have been limited in Europe and North America [35].

Integrated multi-trophic aquaculture systems are an attractive, environmentally sustainable, and circular method of producing several aquatic species [35]. These systems are designed so that farmed finfish or shrimp are raised near extractive species such as macroalgae and shellfish, enabling the recycling of waste from one species as a source of nutrients that compliments the use by another species [35]. These IMTA systems mimic natural marine ecosystems where different species from different trophic levels of the food web are farmed together to complement each other in a balanced and sustainable system [35]. These IMTA systems involve a fish or shrimp species fed commercially formulated feeds; seaweed absorbs carbon, nitrogen, and phosphorus from fish waste; shellfish (e.g., mussels, oysters) remove and filter uneaten fish feed and organic particulates from the water column; and detritivores (e.g., sea cucumbers and polychaetes) remove organic matter (OM) from sediments at the bottom of the water column to reduce waste accumulation and enhance aquatic ecosystem health [35].

Open land-based intensive seaweed cultivation systems (open ponds, raceway ponds, and tanks) allow greater control over cultivation conditions and easier harvesting compared with open ocean systems, have lower capital and operational costs than closed land-based systems (photobioreactors), but have the disadvantages of water evaporation, lack of temperature control, and potential for contamination and yield loss from adverse weather conditions [35]. However, despite the greater capital investment and operational costs of photobioreactors, potential for overheating, contamination and fouling, and extending cleaning and maintenance times, they are useful for providing the amount of control needed to produce sensitive, high value seaweed species used for bioprocessing to produce pharmaceuticals, nutraceuticals, and cosmetics [35].

The environmental impact of the various seaweed production and harvesting systems is highly variable [35]. In the commercial cultivation systems, energy is consumed in the hatchery, preparation of seeding lines, powering pumps, filters, and lights, mechanical harvesting equipment, transportation, drying and bioprocessing [35]. Seaweed is harvested using different methods including manual collection on foot or by boat, mechanized equipment on boats, seaweed cutting vessels, and trawlers which vary in fuel consumption and environmental impact [35]. Currently, there is little if any coordination or influence of decisions among stakeholders within seaweed supply chains, which have limited the opportunity to optimize resources and customize their use for specific market applications [35]. This could include exploiting unique cultivation and harvest conditions to enhance the desired chemical composition and yield of some seaweed species [35].

### 4.4. New Technologies to Enhance Yields, Chemical Composition, and Resilience

New technologies such as drones and sensors can be used to provide real-time data for monitoring weather, water quality, ocean nutrient concentrations are being used to enhance seaweed growth [36]. These technologies can be integrated with artificial intelligence and machine learning to develop algorithms and models to analyze data for detection of diseases, decision support for optimal planting and harvest times to improve yields, reduce labor costs and improve efficiency [37]. Furthermore, use of genetic engineering and selective breeding technologies can produce disease resistant, climate resilient, high yielding new varieties of seaweed with less inputs, with improved nutrient and bioactive compound profiles and concentrations [38]. Genetic selection and engineering can also improve carbon sequestration and maximize space efficiency by growing seaweed in scalable, vertically stacked structures to make seaweed a greater contributor toward mitigating climate change [39].

## 5. Industrial Applications for Seaweed

Several commercially produced and harvested seaweed species provide essential nutrients along with unique and valuable biochemicals that have many industrial applications in human foods, animal feed, pharmaceuticals, cosmetics, biofuels, biofertilizer, biostimulants, biodegradable plastics, packaging material, and waste treatment (Table 2). It is important for readers to understand the competing uses of seaweed beyond animal feed applications because some of these industrial applications such as use as food hydrocolloids, pharmaceuticals, and cosmetics have high economic value in the human market which may affect the economic feasibility of using the same seaweed species and their extracts in animal feeds. Furthermore, some seaweed bioactive compounds with applications in human food and health products may have applications in diets for food producing animals. However, almost all of these applications require specialized processing technologies to overcome extraction challenges caused by its rigid cell walls so that the desired target compounds can be obtained, which also increases the cost of seaweed extracts [40].

The most common uses of several seaweed species are as sources of essential nutrients in human diets and nutraceutical supplements to prevent various health problems, especially in Asian countries [41,42]. Many seaweed species are a good source of dietary fiber, minerals, protein and amino acids, vitamins, and fatty acids (FA) [43], but the nutrient content is highly variable among and within species. Several species of seaweed contain relatively high concentrations of alginate, carrageenan, fucoidan, laminarin, agar, and other phycocolloids that are used as stabilizers, emulsifiers, and thickeners in the food industry [44]. Seaweed is a rich source of vitamins, macro minerals (calcium, sodium), and trace minerals (iodine, zinc, iron, manganese, selenium) which contribute to it being considered a functional ingredient in food [41,42]. Furthermore, a wide range of nutraceutical and bioactive compounds derived from seaweed, such as polyphenols, polysaccharides, bioactive peptides, among others provide numerous health benefits as dietary supplements to humans.

Seaweed has also historically been used in animal feed, especially for cattle, sheep, and goats raised along coastal regions, where it has served as a source of nutrients and phytochemicals that enhance animal health [45]. Because seaweed production does not require land, water, synthetic fertilizer, and other environmentally costly inputs like terrestrial produced animal feed ingredients, it can also reduce the environmental footprint of producing meat, milk, and eggs. However, in general, the high salt, recalcitrant fiber, and water content, moderate to low protein and lipid content, and the potential contamination of heavy metals have generally limited diet inclusion rates to less than 10%, and more commonly to only 1 to 2% of the total diet among all seaweed and food animal species. The numerous phytochemicals (e.g., sulfated polysaccharides, pigments, polyphenols) found in various concentrations among seaweed species provide much greater value in diets for food producing animals than feeding seaweed strictly as a nutrient source. Bioactive compounds in seaweed provide antimicrobial [20,21,46,47], antiviral [20,48,49], antioxidant [21,50], and anti-inflammatory [51] benefits in both human and animal diets. However, extracting and purifying these biochemical compounds requires pre-treatment and efficient separation technologies to maximize yields, preserve bioactivity, and minimize losses [52,53]. More recently, bromoform and halogen compounds present in relatively high concentrations of several red seaweed species, have emerged as another commercially important phytochemical because of their consistent benefits for substantially reducing enteric methane emissions when fed to ruminants [7,54].

Macroalgae extracts are used in several cosmetics and skincare products, such as creams, lotions, and ointments, because of their anti-aging, anti-wrinkle, and moisturizing properties [55,56,57,58]. The vitamins, minerals, FAs, antioxidants, and other bioactive compounds in seaweed are generally highly bioavailable and help reduce redness, blemishes, and skin damage by hydrating, brightening, and re-mineralizing skin tissues [57,59]. Specific bioactive compounds in seaweed also can help reduce hyperpigmentation of skin [60,61], acne vulgaris [62], skin aging [61], and promote skin lightening [55,63].

Other commercial applications of seaweed include using specific phytochemicals for pharmaceuticals and drug development [49,64], production of biofertilizers and soil conditioners that enhance soil health and crop yields [65,66,67,68], making biodegradable plastics [69,70,71,72], use as a feedstock for biofuels production [73,74,75], for environmental cleanup, nutrient removal, and habitat restoration to improve the health of marine environments, and carbon sequestration to minimize climate change impacts [76,77].

## 6. Seaweed Bioprocessing

Seaweed bioprocessing is an essential component of the value chain because it involves extracting multiple bioactive compounds of interest in human and animal health including polysaccharides, polyphenols, minerals, vitamins, pigments, fiber, protein, amino acids, and terpenoids, sterols, polyunsaturated fatty acids (PUFA) [78,79]. In general, *in vivo* and *in vitro* studies evaluating the productivity and health responses from feeding seaweed to food producing animals show more consistent and greater responses when feeding extracts rather than intact seaweed. The lack of standardized seaweed collection can affect the biochemical extraction process, while drying methods, extraction techniques, solvent type and ratio, extraction temperature and duration affect the composition and biological activity of the extract [78]. Therefore, inconsistent responses from feeding the same types and amounts of seaweed extracts can also occur due to the type of extraction methods used which can affect biological activity [80,81].

### 6.1. Post-Harvest Processing

After harvest, marine biomass is washed and cleaned, dried, ground, and sifted into a powder for further processing. However, each of these post-harvest steps pose various challenges. Washing and hot water stabilization removes surface debris and excess iodine, but it also causes unintended losses of water-soluble vitamins, minerals, and bioactive compounds [82]. However, heavy metals and impurities are not removed from simple rinsing because they are embedded in the complex cell wall structures [82]. If seaweed is not washed shortly after harvest, bacteria can multiply rapidly leading to spoilage and degradation of nutritional value [83].

Because fresh seaweed contains up to 90% water, it is highly susceptible to rapid spoilage which requires drying for long-term preservation and economical transport to bioprocessing facilities [84]. Although traditional sun drying is relatively inexpensive, it requires several days or weeks depending on weather conditions and is at greater risk for mold or weather damage, while hot air convection drying dehydrates seaweed much faster but it requires a high amount of energy and degrades the biological activity of heat sensitive nutrients and phytochemicals [83,84,85,86]. Depending on the technology used, drying can represent up to 80% of total seaweed processing costs [84].

The highly fibrous and often rubbery cell walls of semi-dried, and sometimes even dried seaweed can make grinding for particle size reduction difficult without high energy inputs [87]. Standard grinding equipment can generate friction and heat that can degrade heat sensitive lipids, vitamins, and proteins. Seaweed can also retain variable amounts of moisture in pockets of dried fronds which can lead to clogging and uneven particle breakdown during milling.

### 6.2. Extraction Processes

Extraction method determines the specific types and amounts of compounds obtained, their subsequent biological activity, and the environmental impacts of the methods used. Therefore, the methods used to extract phytochemicals, nutraceuticals, and bioactive compounds from seaweed are extremely important because the chemical characteristics (polarity, water or lipid solubility, chemical structure) of target compounds dictate the type of solvent to use (water, ethanol, ethyl acetate), the type of seaweed and cell wall complexity determines if enzyme-assisted or high-pressure extraction is required, and the intended use (food or feed additive, pharmaceutical) determines the required purity, safety standards, and cost-effectiveness [88,89].

Extracting compounds from seaweed begins with pre-treatment processes that include washing, drying, grinding, and sifting to obtain a powder which is further pulverized to obtain a homogeneous substrate. Samples analysis involves numerous types of different procedures depending on the specific chemical compounds of interest. For example, liquid chromatography, ultraviolet and visible spectrophotometry, and Fourier-transform infrared spectroscopy can be used to identify and quantify pigments and phenolic compounds, but (FAs) and amino acids are detected with chromatography [64]. Proteins can be measured with spectrophotometry, and polysaccharides are measured with spectroscopy [64]. Next, isolation and identification of compounds occurs by (1) using pharmacognostic analysis to separate fractions into ether, alcohol, and aqueous extracts, (2) using specific physicochemical analyses to separate total flavonoids and phenols, individual phenols, vitamins, minerals, and pigments, and (3) maceration to produce supernatant to be analyzed using specific physico-chemical analyses for proteins, sulfates, carbohydrates, lipids, and insoluble, soluble, and total dietary fiber [90]. Traditional methods are time consuming, may have safety or toxicity issues depending on the types of solvent used, and negative environmental impacts [91]. Cadar et al. [78] summarized the benefits and limitations of common seaweed extraction methods that include conventional chemical extraction, supercritical fluid extraction, microwave assisted extraction, enzyme assisted extraction, ultrasound assisted extraction, and pressurized solvents extraction (Table 3).

There are several advanced processing and extraction methods including ultrasound-assisted extraction, microwave-assisted extraction, pulsed electric field, subcritical water extraction, subcritical solvent extraction, pressurized liquid extraction, supercritical fluid extraction, high hydrostatic pressure, solid-phase extraction, solid-phase microextraction, enzyme-assisted extraction, osmotic lysis, alkali extraction, pH-shift extraction, and compressed fluid-based extraction that offer many improvements compared with conventional methods [92,93,94]. Many of these technologies increase process yields, provide greater selectivity, reduce processing time, ensure greater biological activity in final extract, and reduce the environmental impact of seaweed products by reducing energy consumption and solvent use, waste, and avoiding hazardous solvents that make them safer and more economical for industrial scale use [95]. The type of bioprocessing method used is also important to protect the biopotency of heat-sensitive or fragile compounds such as polyphenols and peptides that may be degraded under conditions of excessive heat or strong solvents, enhancing purity by targeting specific molecules, and increasing yield and concentration by optimizing conditions to ensure maximum release of compounds [81,94]. However, future development is needed to optimize, automate, and customize adaptable extraction processes for specific industries that utilize low environmental impact solvents and energy sources using standardized protocols to ensure reproducibility and regulatory compliance [93].

### 6.3. Fermentation Processes to Enhance Nutritional Value of Intact Seaweed

Solid state fermentation using fungi, bacteria, and yeast is becoming a cost-effective technology to enhance the nutritional value of by-products by increasing the concentration of essential nutrients (e.g., protein and amino acids) and bioactive compounds (e.g., enzymes, total phenolics) while simultaneously degrading anti-nutritional factors [96]. The high dietary fiber concentrations and recalcitrant polysaccharide structure of seaweed is one of the major factors limiting the use and diet inclusion rates of intact seaweed in livestock and poultry diets. Results from several studies have shown that using several food grade fungal species in solid state fermentation systems are effective in not only degrading polysaccharides, but they can also reduce anti-nutritional factors, improve the nutritional value, and produce high value enzymes and phenolic compounds. *Aspergillus oryzae* can assimilate nutrients in pretreated *Sargassum* spp. to produce fungal proteins while reducing ash and heavy metal concentrations [97]. Red seaweed (*Gelidium*) by-product from agar extraction was co-fermented with sunflower cake, rapeseed cake, or rice bran using *Aspergillus niger* to produce high value enzymes [98]. Several seaweed species (*Gracilaria* sp., *Porphyra dioica*, *Codium tomentosum*, *Ulva rigida*, and *Alaria esculenta*) were fermented with *Aspergillus ibericus* and *Aspegillus niger* to produce high value carbohydrases [99]. Using a submerged fermentation process with a marine fungus (*Paradendryphiella salina*) to ferment *Macrocystic pyrifera* and seaweed waste increased protein and amino acid concentrations, decreased total carbohydrate, alginate, and cellulose content, and increased total phenolic concentrations and antioxidant activity compared with non-fermented biomass [100].

Like fungal fermentation, bacterial fermentation degrades recalcitrant dietary fiber to improve digestibility and prebiotic effects, increases protein quality and digestibility, synthesizes water soluble vitamins and phenolic compounds, reduces anti-nutritional compounds, acidifies, and produces compounds that have anti-inflammatory and antimicrobial properties [96]. For example, using compound strains of lactic acid bacteria for solid state fermentation of *Pyropia* spp. resulted in increased soluble protein concentration, free peptides with expected biological functions, antioxidant activity [101]. Fermenting rapeseed meal and soybean meal along and in combination with *Ascophyllum nodosum* and *Saccharina latissima* seaweed using *Pediococcus acidilactici*, *Pediococcus pentosaceus*, and *Lactiplantibacillus plantarum* releases secondary antimicrobial metabolites to control *Salmonella enterica* Typhimurium, and *Listeria monocytogenes*, *Yersinia enterocolitica*, and *Staphylococcus aureus* in pig feed [102]. Similarly, using lactobacilli to ferment a rapeseed-seaweed blend (*Ascophyllum nodosum* and *Saccharina latissimi*), which was subsequently fed to weaned pigs reduced gut lymphocyte infiltration, improved colon mucosal barrier, and altered gut microbiome composition [103]. Feeding a fermented rapeseed meal-seaweed supplement has also been shown to alter gut microbiota toward a composition that may be associated with improved gut health and may be useful in reducing helminth (*Ascaris suum*) infections in outdoor pigs [104]. Therefore, solid state fermentation technology is a promising and emerging approach to enhance the nutritional value of seaweed and low value seaweed by-products for use in animal feeds.

## 7. Chemical Composition of Seaweed and Extracts

### 7.1. Nutrient Composition

There is large variation in nutrient profiles among seaweed species and sources due to time of collection, habitat, water quality and nutrient concentration, temperature, light intensity. In general, green and red algae species are considered to have greater nutritional value in animal feeds than brown species due to their greater protein and lower mineral concentrations. Seaweed contains high concentrations of carbohydrates which are predominately polysaccharides that result in high dietary fiber concentrations and relatively low energy value for monogastric animals. Dietary fiber concentrations range from 4.8% to 75.7% among green seaweed species [43], 4.8% to 71.4% among brown seaweeds [105], and 5.0% to 53.2% among red seaweed species [106]. Therefore, it is not surprising that the concentration of total carbohydrates ranges from 4% to 76% among macroalgae species on a dry weight basis [107]. The predominant seaweed polysaccharides are laminarin, fucoidans, agar, carrageenans, porphyrin, ulvans, and alginates, which are fermentable in animal digestive systems and provide prebiotic effects [108].

The concentration of protein is also highly variable among seaweed species, ranging from 5.0% to 19.7% in brown seaweed, 3.2% to 29.8% in green seaweed, and 0.67% to 45.0% in red seaweed [43]. Although macroalgae have relatively high protein quality with proportions of essential amino acids comparable or exceeding those of traditional feed ingredients such as corn, rice, wheat, and soybean meal, it also contains relatively high concentrations of non-protein nitrogen, which results in lower nitrogen to crude protein conversion factors compared with traditional terrestrial-based feed ingredients. Furthermore, the concentrations of essential amino acids are substantially less than those found in corn and soybean meal, which limits the use of seaweed as a protein source in monogastric diets but not ruminant diets.

Seaweed contains relatively low concentrations of lipids (1–5%) which are predominantly PUFA [45]. However, these lipids can be a rich source of omega-3 and omega-6 FAs, representing 10 to 70% of total FAs [109]. Some brown seaweed species, such as *Macrocystis pyrifera* contain up to 20% of total lipids as arachidonic acid (20:4 n-5), and up to 18% as acid eicosapentaenoic acid (20:5 n-3); [110]. Eicosapentaenoic acid is an essential omega-3 FA that has potent anti-inflammatory properties, and arachidonic acid (20:4 n-5) is an essential omega-6 FA that is also an anti-inflammatory mediator involved in regulating immune responses.

Due to the ability of seaweed to absorb inorganic substances from the environment, many species contain high ash (mineral) content but varies from 7–40% [45] and can be 10 to 20 times greater than found in terrestrial plants [111,112]. Unfortunately, bioaccumulation of heavy metals (aluminum, arsenic, cadmium, lead, mercury, nickel, strontium, and tin) can also occur [113]. Seaweed contains relatively high concentrations of nutritionally essential macro minerals (calcium, phosphorus, sodium, potassium), along with essential micro minerals (copper, iron, iodine, manganese, selenium, zinc), but iodine concentrations are generally very high, with much greater concentrations found in brown seaweed (1200 mg/100 g dry matter; DM) than in green (2–25 mg/100 g DM) and red (10–100 mg/100 g DM) seaweed [114]. The high iodine, sodium, and potentially arsenic concentrations are major factors limiting diet inclusion rates in animal feds to avoid toxicity.

Algae are excellent sources of fat-soluble vitamins (A, D, and E) and water-soluble vitamins (B_1_, B_12_, C, riboflavin, niacin, pantothenic acid, and folic acid) which contribute to many of the improved health responses attributed to feeding seaweed to livestock and poultry. In addition, seaweed contains relatively high concentrations of pigments, which vary among seaweed species [115], but have potent antioxidant, antimicrobial, and anti-inflammatory activities [116,117,118]. Pigments derived from seaweed are generally classified as carotenoids, chlorophylls, and phycobiliproteins [118]. The main pigments in green seaweed are chlorophyll a and chlorophyll b, and some *β*-carotene and lutein. Pigments in red seaweed are phycoerythrin, phycocyanin, *β*-carotene, lutein, zeaxanthin, and chlorophyll, while brown seaweed contains fucoxanthin, *β*-carotene, violaxanthin, diatoxanthin, and chlorophyll.

### 7.2. Bioactive Compounds

In addition to the nutrients that seaweed can provide to animal diets, they also provide polysaccharides, phenolic compounds, bioactive peptides, and pigments that provide prebiotic, antimicrobial, antioxidant, anti-inflammatory, and immune modulating properties that enhance human and animal health. Comprehensive reviews have summarized the numerous nutraceutical and bioactive compounds in many types of seaweed [8,18,45,78,119,120,121,122], with some reviews specifically focusing specifically on brown seaweed [123,124,125,126], green seaweed [127,128], and red seaweed [129,130,131]. Several reviews have specifically focused on feeding intact seaweed and extracts to ruminants [132,133], swine [108,134,135,136], and poultry [135,137,138,139,140]. A summary of the numerous human and animal health benefits derived from various bioactive compounds and nutraceuticals obtained from target commercially available seaweed species is shown in Table 4.

#### 7.2.1. Sulfated Polysaccharides

Sulfated polysaccharides (fucoidan, alginate, carrageenan, and agar) are well documented to have antioxidant, antiviral, anticoagulant, anti-inflammatory, and immunomodulatory activities [49,79,141]. Polysaccharide composition varies among brown (fucoidan, laminaran, alginates, cellulose), green (ulvan, starch, xyloglucan, cellulose, hemicellulose, xylopyranose), and red (agar, cellulose, carrageenan, porphyrin, agaropectin, xylan, galactans, mannans) seaweed species [141,142]. Concentrations (dry weight basis) of polysaccharides also vary widely within and among major brown (*Ascophyllum* = 40–70%, *Fucus* = 62–66%, *Laminaria* = 38–68%, *Sargassum* = 49–62%, *Undaria* = 35–46%), green (*Ulva* = 15–65%), and red (*Chondrus* = 55–66%, *Porphyra* = 40–54%, *Gracilaria* = 36–63%, *Palmaria* = 38–74%) genera [142]. Fucoidans, laminarin, alginates, and phlorotannins found in brown seaweed species provide potent antioxidant (phlorotannins) and anti-inflammatory (fucoidans) activities, while ulvan in green seaweed provides immune and antioxidant benefits and the agar, porphyrin, and galactans in red seaweed provide prebiotic and gut health benefits [45,134,143]. Greater amounts of attached sulfate groups and lower molecular weights increase antioxidant effectiveness of seaweed polysaccharides [144].

The abundant amount of polysaccharides found in seaweed also serves as a source of fermentable dietary fiber that provides prebiotic effects. Prebiotics are carbohydrates that are indigestible to hydrolytic enzymes but are fermentable in the lower gastrointestinal tract of animals [145]. Prebiotic effects are generally evaluated based on increases in short chain FAs acid production, decreases in opportunistic pathogenic bacteria (e.g., *Escherichia coli*, *Salmonella*), and increases in beneficial bacteria (*Lactobacillus*) in the intestinal tract and feces [145]. Therefore, these undigested fermentable polysaccharides and oligosaccharides alter the intestinal microbiome composition by stimulating growth of *Lactobacilli* and *Bifidobactera* (beneficial bacteria), metabolism, and fermentation, and can reduce the adhesion and populations of pathogenic bacteria such as *Escherichia coli* and *Salmonella typhimurium* [20,136,146]. Several studies have shown that brown seaweed polysaccharides derived from *A. nodosum*, *L. digitata*, *L hyperborea*, and *Laminaria* spp. can be effective in reducing enteric pathogens in growing-finishing pigs [134]. Laminarin and fucoidan are effective in reducing fecal enterotoxigenic *Escherichia coli* count and increasing *Lactobacillus* growth which can reduce post-weaning diarrhea and improve gut health, growth rate, and feed conversion [147,148]. Fucoidan enhances *Lactobacillus* growth in the intestinal tract which can lead to improved nutrient digestibility, and feeding these prebiotics from *Ascophillum nodosum* increased short chain FAs and butyric acid from carbohydrate fermentation improved gut health [149,150]. Alginic acid oligosaccharides have antibacterial activities that can improve intestinal barrier function, growth of *Lactobacillus*, and alter antibody production [151,152].

#### 7.2.2. Polyphenols

Compared with grain- and oilseed-based feed ingredients, seaweed is a rich source of natural antioxidants and antimicrobials. The main types of antioxidant compounds found in seaweed are phenolics, polysaccharides, and pigments. Phenolic compounds include simple phenols classified as hydroxycinnamic acids (e.g., caffeic, *p*-coumaric, ferulic, sinapic; [153]), hydroxybenzoic acids (e.g., gallic, vanillic, 4-hydroxybenzoic, protocatechuic, syrinic, gentisic; [154]), and polyphenols that include flavonoids and non-flavonoids. Flavonoids include flavones, flavanol, flavanones, flavonols, anthocyanins, and isoflavones, while non-flavonoids include tannins (e.g., phlortannins), lignans, and stilbenes [129,142,153,154]. Other types of polyphenols found in seaweeds include terpenes, terpenoids, and bromophenols [129,142,153]. In addition to antioxidant properties, most phenolic compounds also provide antibacterial, antiviral, anti-inflammatory, and anti-methanogenic activities [108,129,155].

#### 7.2.3. Pigments

Pigments derived from seaweed have antioxidant properties [112,114,153,156], and are generally classified as carotenoids, chlorophylls, and phycobiliproteins [118]. The main pigments in green seaweed are chlorophyll a and chlorophyll b, and some *β*-carotene and lutein. Pigments in red seaweed include phycoerythrin, phycocyanin, *β*-carotene, lutein, zeaxanthin, and chlorophyll, while brown seaweed contains fucoxanthin, *β*-carotene, violaxanthin, diatoxanthin, and chlorophyll. In addition to their antioxidant properties, pigments also have immunomodulatory, anti-diabetic, anti-cancer, anti-inflammatory, and anti-angiogenic properties [157].

#### 7.2.4. Bioactive Peptides

Seaweed proteins consist of several valuable bioactive and functional components including specific enzymes, glycoproteins, phycobiliproteins, lectins, and peptides [158]. However, because of the complexity and variation in composition of seaweed cell walls, pre-treatment, extraction, and purification techniques must be carefully selected to avoid undesirable disruptions during enzymatic, physical, and chemical treatments during the hydrolyzation process to maintain bioactive properties of hydrolysates [158]. Bioactive properties of seaweed protein hydrolysates and peptides include antioxidant, anti-inflammatory, antimicrobial, antitumor, anticoagulant, anti-hypersensitive, and immunostimulatory activities [158,159]. Bioactive peptides derived from seaweed can be used as therapeutic drugs and functional nutrients for preventing cardiovascular disease and diabetes in humans. Several studies have reported that peptides obtained from seaweed have angiotensin converting enzyme inhibition, and antihypertensive, antioxidative and antidiabetic activities [160]. Although nearly all of the studies have focused on the application of bioactive peptides in seaweed in humans, there are significant opportunities for using specific types that provide antioxidant, anti-inflammatory, antimicrobial, and immune stimulatory activities in food producing animals. However, further development is needed to efficiently isolate and extract peptides in large-scale production facilities and validate their health benefits in *in vivo* studies [159,160].

#### 7.2.5. Bromoform and Other Polyhalogenated Compounds

Because of the enormous need to reduce greenhouse gas emissions from food animal production, and the relatively high proportion of these emissions attributed to enteric methane emissions from beef and dairy cattle, a significant amount of research has been devoted to showing that bromoform and other polyhalogenated compounds (bromochloromethane, chloroform, and iodoform) derived from brown seaweed has a dramatic effect on reducing enteric methane emissions when fed to ruminants [161,162]. As a result, a plethora of recent reviews have focused on the use of seaweed and extracts containing bioactive components on mitigating methane and greenhouse gas emissions in ruminants [54,163,164,165,166,167,168]. Bromoform is the primary compound found in red seaweeds such as *Asparagopsis* genus and is a halogenated methane analogue that interferes or inhibits the function of enzymes, especially methyl-coenzyme M reductase (MCR) in the methanogenesis pathway when 1–2% seaweed is added to the diet [161]. Other compounds including tannins, polyphenols, and prebiotic oligosaccharides also have been shown to reduce methane emissions [54]. As a result, although bromoform is the most potent anti-methanogenic compound, feeding an equivalent dose from the whole seaweed biomass resulted in 36–40% greater methane reduction than feeding bromoform alone [169]. Therefore, to achieve the same amount of methane reduction from feeding whole seaweed, a higher dose of bromoform is needed which has potential toxicity and safety concerns for animals and humans, which is unstable and volatile [170]. A summary of the effects of seaweed and other bromoform-containing feed ingredients on methane production, yield, and intensity in cattle [168]. Bovaer is a commercially available feed additive that contains 3-nitrooxypropanol (3-NOP) that inhibits MCR and has been shown to reduce methane emissions by 30 to 45% in cattle [171], is metabolized and does not carry over into meat and milk, is safe for animals, producers, and consumers, and therefore, is approved for use in the EU [172], Brazil, and US (for lactating dairy cows). However, feeding whole seaweed (*Asparagopsis armata*) containing bromoform has been shown to reduce methane emissions up to 95% in many studies compared with feeding Bovaer or synthetic bromoform [169]. However, although seaweed-derived bromoform has been shown experimentally to be effective in substantially reducing enteric methane emissions from ruminants, energy intensive processing and long transport distances can offset methane abatement gains [7]. Therefore, optimizing algae sourcing, processing, and application at a larger scale is needed to achieve the full potential of seaweed’s contribution to reducing the environmental burden of ruminant production systems.

## 8. Chemical Analysis Challenges of Seaweed

There are numerous analytical challenges for determining the nutrient and bioactive content of seaweed including complex cell wall structures, low solubility, molecular similarities between target and non-target compounds, high natural variability in biochemical composition, and structural vulnerability and degradation [122,173]. Low solubility of some compounds such as proteins requires enzymatic or chemical pre-treatments to break the cell wall before analysis [173,174]. Seaweed cell walls contain complex, rigid, highly cross-linked polysaccharides, such as alginate, carrageenan, and fucoidan, that trap nutrients within this matrix and result in the need to use extensive enzymatic pre-treatments and harsh extraction methods that can degrade heat sensitive bioactive compounds [78,173,175,176,177]. Extraction efficiency of polysaccharides (alginate, aga, carrageenan, and ulvan) is relatively low and ranges from about 2.5% to 14.2%, with slightly greater (5.3% to 27.1%) extraction efficiency reported for bioactive compounds among five different algal species [178].

Many compounds such as polyphenols, antioxidants, pigments, and complex carbohydrates can mask results or interfere with spectroscopic and chromatographic detection [179,180], especially with the detection of target proteins and peptides [79,173]. As a result, high concentrations of salts, minerals and complex carbohydrates are often included in extracts of target bioactive molecules such as proteins and phenolics, which require extensive purification before quantification [173,175,181].

Standard nitrogen-to-protein conversion factors such as the widely used nitrogen (N) × 6.25 for estimating the crude protein content of seaweed are inaccurate because a substantial portion of the total N in seaweed is derived from non-protein sources such as nucleic acids and free amino acids [173,182,183]. Furthermore, although amino acid hydrolysis is a standard method for breaking down proteins to amino acids to determine amino acid profiles of feed matrices, it is a harsh process that partially or completely destroys some amino acids such as tryptophan, cysteine, and methionine [79].

Many bioactive compounds such as some vitamins (vitamin E), pigments (fucoxanthin), and unsaturated FAs (omega-3) are very sensitive oxidation when exposure to heat, light, and oxygen. Conventional lipid extraction methods such as Soxhlet, degrade these compounds before they can be measured [79,95,175,184].

The nutrient and bioactive content of seaweed is highly variable based on species, harvesting season, geographic location, water temperature, and light exposure. As a result, it is difficult to establish standardized baseline nutritional profiles for seaweed compared with terrestrial grown crops [127,185,186].

To overcome these challenges, researchers are transitioning from conventional analytical methods to advanced, “green” analytical techniques such as microwave-assisted extraction, supercritical fluid extraction, and enzyme-assisted extraction to increase extraction yield and minimize damage to the structural integrity of thermolabile compounds. Amino acid analysis is the recommended reference method to assess the true protein content of seaweed instead of Kjeldahl methodology to determine nitrogen content and calculate crude protein estimates [79,95,176].

## 9. Effects of Seaweed and Extracts in Ruminant Diets

### 9.1. Lactating Dairy Cows

Variable responses have been reported on the effects of feeding intact seaweed, blends, and extracts to lactating dairy cows on milk yield, milk composition, nutrient digestibility, rumen fermentation, and enteric methane emissions. This is not surprising because of the wide variation in nutritional composition, digestibility, and concentrations of bioactive compounds among seaweed species. Two studies have evaluated the protein digestibility and rumen degradability of various seaweed species fed to dairy cows. Tayyab et al. [187] evaluated the in situ ruminal and intestinal protein degradability of 8 different seaweed species (*Acrosiphonia* sp., *Alaria esculenta*, *Laminaria digitata*, *Mastocarpus stellatus*, *Palmaria palmata*, *Pelvetia canaliculata*, *Porphyra* sp., and *Ulva* sp.) collected during spring and autumn seasons, and reported that *Porphyra* and *Ulva* can provide a high amount of digestible protein to the small intestine, *Acrosiphonia*, *Alaria*, and *Laminaria* can supply a high amount of rumen degradable protein, and *Mastocarpus* and *Palmaria* provide equal amounts of degradable protein to the rumen and small intestine. Similarly, Gaillard et al. [188] determined the in situ degradability of amino acids in nine seaweed species (red—(*Mastocarpus stellatus*, *Palmaria palmata*, *Porphyra* sp.; brown—*Alaria esculenta*, *Laminaria digitata*, *Pelvetia canaliculata*, *Saccharina latissima*; green—*Cladophora rupestris*, *Ulva* sp.) in dairy cows. Results showed that the relatively high amount of amino acid concentrations and degradability, *Porphyra* sp. followed by *Palmaria palmata, Ulva* sp. and *Cladophora rupestris* were determined to be acceptable sources of protein for ruminants, while *Laminaria digitata* and *Mastocarpus stellatus* are good sources of bypass protein with amino acids protected against rumen degradation.

Conducting meta-analysis studies is a useful statistical method for quantitatively aggregating and summarizing variable results from previous studies to resolve conflicts between studies and develop a more definitive conclusion of the effects of feeding seaweed to food producing animals. Although two meta-analyses showed that feeding seaweed can mitigate enteric methane emissions without adversely affecting productivity, these meta-analyses [132,189] only included a limited number of studies, involving a limited number of response variables (*n* = 5; [132]), or combined some responses from multiple ruminant species [189]. Orzuna-Orzuna et al. [133] conducted a more extensive meta-analysis of results from 23 scientific articles and reported that dietary supplementation with seaweed (primarily *Ascophyllum nodosum* and *Asparagopsis taxiformis*) reduced DM intake, milk protein content, milk urea nitrogen, and somatic cell count, and increased milk fat, lactose, and iodine content. Furthermore, feeding diets containing seaweed to lactating dairy cows increased ruminal pH and concentrations of butyrate and valerate, reduced ruminal ammonia nitrogen concentration, acetate to propionate ratio, daily methane emission, yield, and intensity. However, no effects were observed from seaweed supplementation for nutrient digestibility, total volatile fatty acids (VFAs), acetate, and propionate concentrations. Interestingly, results from another study involving feeding diets contain 4% *Himanthalia elongata* or 4% seaweed extract as a source of phlorotannins for 22 days to lactating dairy cows resulted in a 46% and 28% reduction, respectively, in ammonia emissions from slurry [190], which is in agreement with reduced ruminal ammonia concentrations from the meta-analysis of Orzuna-Orzuna [133]. Unfortunately, other studies involving diet supplementation of other types of seaweed or seaweed extracts were not included in this meta-analysis [133]. Lastly, two studies with preweaning dairy calves have shown that feeding diets containing 5% of either *Ascophyllum nodosum*, *Ulva lactuca*, or *Saccharina latissima* (DM basis) for 40 days had no effect on growth performance or gut health but increased innate immune response [191,192]. A brief summary of nutritional and performance responses from feeding various intact seaweed species, extracts, and blends is shown in Table 5.

### 9.2. Beef Cattle

A limited number of studies have evaluated the effects of feeding selected species of intact seaweed and commercial products and blends in beef cattle. A meta-analysis study based on 25 *in vitro* and 22 *in vivo* studies involving dairy, beef, and small ruminants showed that *Asparagopsis taxiformis* had the greatest reduction (65%) in methane emissions among seaweed species evaluated, an interaction between dietary levels and species for the proportions of acetate, propionate and butyrate production, but had no effect on dry matter intake and growth rate compared with feeding control diets [189]. Similarly, Lean et al. [132] conducted a meta-analysis involving 14 experiments and reported that *Asparagopsis taxiformis* and *Ascophyllum nodosum* were the main seaweeds evaluated and results indicated no differences in average daily gain (ADG) and DMI, but cattle fed *A. toxiformis* had greater improvements in feed efficiency. These researchers indicated that more *in vivo* studies are needed to evaluate the practical applications and potential risks of feeding seaweed to beef and dairy cattle [132]. Most studies that analyzed carcass muscle, fat and organs for bromoform concentrations reported non-detectable or concentration of negligible risk for human health. Although bromoform is considered to be a probable carcinogen in high doses, very little accumulates in animal tissues resulting in negligible food safety risk [222]. A brief summary of growth performance, methane emissions, and carcass quality responses from feeding various species of intact seaweed, extracts, and blends to beef cattle is shown in Table 6.

### 9.3. Dairy and Meat Goats

Only a few studies have evaluated the performance responses of feeding seaweed to dairy and meat goats. Romero et al. [241] observed that feeding a combination of 2% *Asparagopsis taxiformis* and phloroglucinol reduced methane production, prevented accumulation of rumen dihydrogen, increased acetate and total VFA production to enhance rumen fermentation in Murciano-Granadina adult goats. Results from another study showed that feeding diets up to 12% DMI had no effect on DMI and milk production of Saanen dairy goats subjected to heat stress but reduced respiration rate, rectal temperature, and surface temperature indicating the heat stress in dairy goats can be alleviated by feeding diets containing the red seaweed *Gracilaria birdiae* [242]. Growing meat-type goats subjected to transportation stress preslaughter had improved antioxidant status from diet supplementation of 2% seaweed extract from *Ascophyllum nodosum* [243]. However, results from another study showed that body weight loss and changes in blood metabolites resulting from transportation stress varied between goat breeds but were not affected by feeding a diet supplemented with *A. nodosum* extract [244]. Supplementing meat goat diets with *A. nodosum* extract 14 days before slaughter and using a spray wash of chlorinated water during processing reduced *E. coli* counts [245].

### 9.4. Sheep

Several studies have been conducted to evaluate nutritional and performance responses from feeding intact seaweed and seaweed extracts to mature sheep and lambs. However, only a limited number of brown (*Ascophyllum nodosum*, *Laminaria digitata*, *L. hyperborea*, *Saccharina latissimi*, *Sargassum* spp.), green (*Ruppia maritima and Chaetomorpha linum*), and red (*Asparagopsis taxiformis*, *Porphyra* spp.) species have been evaluated (Table 7). Results from a meta-analysis study involving the effects of feeding dairy and beef cattle and small ruminants diets supplemented with various seaweed species showed that *Asparagopsis taxiformis* had the greatest effect in reducing methane emissions in beef cattle, but the effect was non-significant in dairy cows and small ruminants compared with feeding control diets [189].

**Table 7:** Summary of responses from studies evaluating dietary inclusion of selected seaweed species on nutritional and performance responses in sheep.

Seaweed Species	Responses	References
Brown seaweed		
*Ascophyllum nodosum*	Feeding to gestating ewes interfered with passive immunity, increased mortality rate, but had no effect on adaptive immunity of offspring; increased total rumen VFA production but had no effects on DMI, ADG, G:F	[246,247]
*Laminaria digitata* and *L. hyperborea*	Moderately good (72–80%) OM and DM digestibility is a suitable energy source for a rare sheep breed, but feeding lambs 2% *Laminaria hyperborea* by-product reduced ADG and had no effect on FI and methane emissions	[248,249]
*Saccharina latissima*	Reduced dietary protein digestibility	[250]
*Sargassum* spp.	Feeding 25% DMI to ewes post-weaning had no effect on FI or ADG, but feeding up to 30% DMI increased NDF and ADF digestibility, rumen pH, and deceased rumen VFAs	[251,252]
Green seaweed		
*Ruppia maritima* and *Chaetomorpha linum*	Diet inclusion of 20% of either seaweed had no effect on growth performance, DM, OM, and N digestibility; increasing inclusion of *Ruppia* sp. increased FI, reduced OM digestibility, and had no effect of ADG and N retention whereas increasing diet level of *Chaetomorpha* sp. had no effect on FI, ADG, and N retention but improved OM digestibility	[253,254]
*Ulva* sp.	Increasing dietary levels had no effect on FI, ADG, N retention but decreased OM digestibility	[254]
Red seaweed		
*Asparagopsis taxiformis*	Diet inclusion up to 3% reduced methane production up to 80%, decreased total VFAs and acetate and increased propionate in the rumen with no effect on ADG; feeding up to 45 mg seaweed-vegetable oil immersions per kg of feed reduced methane emissions by 75%, reduced growth performance, caused inflammation in rumen wall but no bromoform resides were detected in meat	[189,255,256]
*Porphyra* spp.	Dietary CP digestibility and ADG were comparable to feeding soybean meal supplemented diets but feeding had no effect on methane emissions	[250,257]
Commercial extracts and blends		
Commercial extract from *Ascophyllum nodosum*	Dietary extract had no effect on growth performance, carcass characteristics but feeding extract treated endophyte infested tall fescue hay to lambs improved OM digestibility, had no effect on N digestibility, and meat sensory characteristics; total ruminal *E. coli* and fecal shedding were reduced by supplementing 3% and 5% of diets without altering rumen fermentation and metabolism	[234,258,259]
1% seaweed meal	Increased FI and hot carcass weight but reduced ADG, and non-carcass fat	[260]

### 9.5. In Vitro Studies to Evaluate Methane Emissions in Ruminants from Seaweed

As previously discussed, the addition of various seaweed species and their extracts to ruminant diets to reduce enteric methane emissions has been the focus of numerous *in vitro* and *in vivo* studies. However, inconsistent responses have been observed among numerous *in vitro* studies. A total of about 60 brown seaweed species has been evaluated for their effectiveness in reducing methane emissions, with 42 studies reporting reductions in emissions and 63 studies showing no effect among these species (Table A1). As expected, fewer green seaweed species (*n* = 27) have been evaluated for their effects on reducing methane emissions *in vitro* because of their generally low concentrations of polyhalogens, and only 9 studies showed reductions in methane emissions compared with 31 studies that showed no effect (Table A2). In contrast, many red seaweed species are known to contain relatively high concentrations of bromoform and about 71 different red species have been evaluated, where 61 studies reported reductions in methane emissions and 58 studies reported no response among these species (Table A3). The lack of methane reduction responses observed among a high proportion of these studies is likely a result of either low concentrations of polyhalogens, low bioactivity in specific seaweed species evaluated, low dose of bromoform tested, analytical challenges, or inadequate methodologies and level of detection. However, this summary of responses indicates that continued research and development of effective bioprocessing methods are needed to support commercial use of bromoform and associated halogens from seaweed in this emerging segment of the seaweed market. Although *in vitro* studies are less costly screening approached to identify candidate seaweed species that are effective in reducing enteric methane emissions, *in vivo* studies are needed to validate *in vitro* reductions in methane emissions by accounting for potential interactions with nutrients, other bioactive compounds, and metabolites in the rumen of different ruminant species. Therefore, *in vivo* studies are essential for further market development of seaweed use in methane emission mitigation in ruminant production systems.

### 9.6. In Vivo Studies to Evaluate Methane Emissions from Feeding Seaweed to Cattle

Compared with the large number of *in vitro* studies that have been conducted to evaluate methane emissions from seaweed, fewer *in vivo* studies (*n* = 32) have been conducted to directly evaluate the effects of feeding a limited number of seaweed species and extracts on enteric methane emissions in ruminants (Table A4). Most of the studies that evaluated six different types of brown seaweed generally reported no effect on methane emissions except for studies by Katwal et al. [202] and Bošnjaković et al. [197]. There have been no *in vivo* studies conducted to evaluate methane emissions from feeding green seaweed species to ruminants. In contrast, the red seaweed species of *Asparagopsis taxiformis* and *Asparagopsis armata* were the predominant species that have been evaluated and results consistently showed reductions in enteric methane emissions across multiple studies. However, the other red seaweed species (*Mazzaella japonica*, and *Porphya* sp.) that were evaluated either had no effect on enteric methane emissions, or responses were inconsistent (*Chondrus crispus*).

## 10. Effects of Feeding Seaweed and Extracts to Swine

There have been a greater number of studies conducted to evaluate the addition of several species of intact seaweed, extracts, and commercial blends in swine diets on reproduction, lactation, and growth performance, health, and meat quality than the number of studies previously summarized for dairy and beef cattle, dairy and meat-type goats, and sheep. As a result, our understanding of the many beneficial effects that dietary seaweed can provide in swine production systems is better understood.

The nutritional composition of many seaweed species are inferior to traditional feed grains, grain by-products, and oilseed by-products used in swine diets. Brown seaweed species generally have low and highly variable protein (7.6–12.6% DM) and lipid (0.8–6% DM) content, and high ash content (19.3–27.8% DM); green seaweed species have slightly greater protein (16.2% DM) but low lipid (1.3% DM) and high ash content (19.3–27.8% DM), while red seaweed species have moderate protein (16.9% DM) and lipid (8.9% DM) content and high ash content (19.3–27.8% DM) [108]. Therefore, diet inclusion rates of seaweed as a nutrient source are limited due the need for relatively high energy and nutrient density in swine diets.

### 10.1. Reproduction

The majority of studies evaluating the addition of seaweed extracts to sow diets were conducted from the last trimester of gestation through lactation and focused on health and performance benefits of offspring. Supplementing sow diets with a seaweed powder containing laminarin and fucoidan [261], seaweed derived polysaccharides [262], and seaweed extract [263] enhanced immune response and gut morphology of suckling pigs. Other studies showed benefits of feeding seaweed extracts to sows which resulted in improvements in gut health by altering the microbiome of sows [264], reducing *Escherichia coli* populations in the intestinal tract and feces of piglets and generally improving growth rate post-weaning [263,265,266,267,268]. However, results from other studies evaluating the prebiotic responses from feeding *Laminaria* sp. to sows during late gestation through lactation and showed variable responses [263,265,267,268,269,270]. Nguyen et al. [271,272,273] evaluated the effects of adding seaweed supplements to low omega-6:omega-3 sow diets and observed no improvement in growth rate or oxidative status of offspring [271]. However, feeding seaweed diets reduced inflammation severity [273] and enhanced host defense during inflammation [272].

A few studies reported improvements in the total number [274] and number of piglets born alive per litter [275,276], increased sow FI [276], improved milk yield and composition [275,276], and litter weight gain [274,276], and offspring growth rate after weaning and throughout the grower-finisher period [277] when supplementing sow diets with different seaweed types or extracts. However, two other studies showed no improvement in milk composition or litter weight gain when seaweed was supplemented only during lactation [278], or inconsistent sow and litter responses when a commercial mixture of brown, red, and green seaweed was fed during gestation, lactation and nursery-finisher phases [279]. As a result, it may be necessary to use longer term supplementation of dietary seaweed during the entire reproductive cycle beginning with developing gilts, followed by gestation and lactation diets to allow an improved microbiome to be established to optimize reproduction, lactation, and litter performance. Dietary changes and the effects of dietary fiber can require several weeks or months of continuous feeding for the gut microbiome to adapt and stabilize [280,281,282,283,284]. Lastly, only one study has been conducted to evaluate supplementing seaweed derived alginate oligosaccharides, which were fed to mature boars for 9 weeks and resulted in increased sperm motility, sperm proteins, and semen volume [285].

### 10.2. Growth Performance

Most growth performance studies have evaluated the addition of intact seaweed and seaweed extracts in weaned pig diets at inclusion rates <5% due to interest in obtaining benefits from their bioactive components rather than their nutritional value. However, studies have generally shown improvements in gross energy, OM, DM, N, NDF, or ash digestibility from feeding brown seaweed (*Ascophyllum nodosum*, *Ecklonia cava*, and *Laminaria* sp.) to weaned pigs [108]. A few studies have specifically evaluated the use of calcitic seaweed (*Phymatolithon calcareum* or *Lithothamnium calcareum*) as a digestible source of calcium in sows [275] and weaned pig diets [286,287], which is of questionable nutritional value because of the low cost and abundance of inorganic calcium supplements available.

Gardiner et al. [288] fed diets containing up to 0.9% *Ascophyllum nodosum* extract to growing-finishing pigs and observed a reduction in growth rate and carcass yield but no effect on FI and G:F. Bouwhuis et al. [289] fed laminarin and fucoidan extract from *Laminaria* sp. and observed an increase in growth rate and slaughter weight but a decrease in FI and G:F.

Most weaned pig studies involved the addition of *Laminaria* sp. because of their relatively high concentrations of laminarin and fucoidan oligosaccharides and have consistently shown beneficial prebiotic effects and improvements in growth rate [290,291,292,293,294]. Studies evaluating the addition of other brown seaweed species and extracts have also shown improvements in growth rate of weaned pigs [295,296,297,298], but not in every case [299].

### 10.3. Health

Although macroalgae species vary widely in their chemical composition, they contain numerous prebiotic, antioxidant, antibacterial, anti-inflammatory, and immunomodulating bioactive compounds that improve swine health and productivity [108]. Combinations of these effects are commonly observed for some types of seaweed. For example, Jia et al. [300] showed that feeding diets containing 0.1% *Gracilaria lemaneiformis* polysaccharide altered the gastrointestinal tract microflora and metabolites to improve antioxidant, anti-inflammatory, and immune responses in finishing pigs.

Prebiotics consist of complex carbohydrates (polysaccharides, oligosaccharides) that are resistant to degradation by hydrolytic enzymes in the intestinal tract but are fermentable, which results in the production of short-chain FA (i.e., butyrate) in the cecum and colon. The primary polysaccharides that have been evaluated for their prebiotic effects in pig diets are laminarin, fucoidan, and alginates in brown seaweed, ulvan in green seaweed, and carrageenan in red seaweed. Macroalgae polysaccharides and oligosaccharides have a significant effect on intestinal microbiome composition by stimulating growth of *Lactobacilli* and *Bifidobactera* (beneficial bacteria), metabolism, and fermentation and can reduce the adhesion and populations of pathogenic bacteria such as *Escherichia coli* and *Salmonella typhimurium* [20,146,301]. Variable responses to changes in cecal, colon, and fecal *Lactobacilli*, *Bifidobacterium*, *Enterobacteriaceae*, *E. coli*, and short chain FA concentrations from feeding brown seaweed alginates [296,302], *Ascophyllum nodosum* extract [150], *Laminaria* sp. extracts [147,291,292,294,303,304,305,306,307,308], intact *Laminaria* sp. [309], intact *Ecklonia cava* [310], and intact *Ascophyllum nodosum* [295,299] have been observed in weaned pigs. *In vitro* studies have shown strong antibacterial and prebiotic effects of *Laminaria* sp. and their extracts for use in weaned pig diets to alleviate gastrointestinal dysbiosis [311]. Additional studies in growing-finishing pigs have also shown variable prebiotic responses from feeding extracts or intact *Laminaria digitata* [312,313,314,315], *Laminaria hyperborea* extracts [312,316], *Laminaria* sp. extracts [270,317], and dried intact *Ascophyllum nodosum* [288].

Several studies have reported improvements in various antioxidant biomarkers in weaned pigs which were often associated with improvements in growth performance [151,296,297,299], and benefits in growing pigs that led to reduced lipid oxidation and improved shelf life of fresh pork [318,319,320]. Frazzini et al. [321] showed that seaweed-derived mixed extracts were effective in enhancing oxidative barrier and immune benefits on porcine alveolar macrophages.

Several studies have shown antibacterial effects of red (*Callophycus serratus*, *Delisea pulchra*, *Pterocladia capillacea*, *Sargassum swartzii*, *Solieria filiformis*, *Sphaerococcus coronopifolius*) and brown (*Ascophyllum nodosum*, *Eisenia bicyclis*, *Laminaria hyperborea*, *Saccharina longicruris*) macroalgae species for several common bacterial strains (*Escherichia coli*, *Salmonella typhimurium*, *Staphylococcus aureus* and *S. aureus* methicillin-resistant, *Campylobacter jejuni*, *Enterococcus faecium* vancomycin-resistant, *Listeria monocytogenes*, *Pseudomonas aeruginosa*, and *Propionibacterium* which are of concern for swine health and pork safety [108]. Bouwhuis et al. [270,322] showed that supplementing sow diets with laminarin reduces fecal shedding of *Salmonella typhimurium* and improves gut health of weaned pigs. Similarly, McDonnell et al. [317] showed that feeding seaweed-derived polysaccharides reduced intestinal and fecal *Salmonella typhimurium* numbers after an oral challenge in grower-finisher pigs, but Sweeney et al. [304] observed increased fecal shedding of *Salmonella typhimurium* at some sampling periods during their challenge study with weaned pigs. In contrast, Turner et al. [323] observed no benefit adding *Ascophyllum nodosum* extract to weaned pig diets and challenged with *Salmonella typhimurium*. In addition, Heim et al. [268] showed a reduction in fecal shedding of enterotoxigenic *Escherichia coli* K88, improved gut health and growth rate when maternal diets were supplemented with *Laminaria* extract. Shimazu et al. [324] supplemented growing-finishing pig diets with Wakame seaweed powder and reported no improvement in growth performance during the growing period, but growth rate was improved during the finisher phase, and the intestinal microflora was altered by increasing *Lactobacillus* and decreasing *Escherichia coli*. The objective of many studies has been to compare the use of seaweed or seaweed extracts as replacements for pharmacological levels of zinc oxide (ZnO) in nursery diets, which have generally shown comparable growth performance and gut health benefits [145,307,325,326,327,328], and when fed in combination with fermented rapeseed meal diets [329,330]. Of the few studies where no improvement in growth performance was observed, diet inclusion rate [331] or administration method [332] may have been factors causing the lack of response.

Most studies evaluating the anti-inflammatory properties of feeding brown seaweed (e.g., *Laminaria digitata*, *Laminaria hyperborea*, *Laminaria* sp.) to pigs have shown positive responses [151,263,267,270,294,296,306,307,314,317,322]. Studies in sows [263,333], weaned pigs [261,263,296,298,333,334], and growing pigs [334] have generally shown improvements in immune function when feeding intact seaweed and extracts. Berri et al. [335] provided *in vitro* derived evidence to suggest that Ulven could be used to complex and deliver Toll-like receptor ligands to relevant target immune cells as part of vaccine strategies, and Hervet et al. [336] showed that oral supplementation with *Ulva*-derived seaweed extracts enhanced immune responses to Porcine Reproductive and Respiratory Syndrome Virus vaccination in pigs. Bonde et al. [337] fed diets containing 8% fermented *Saccharina latissima* to pigs infected with *Ascaris suum* and *Oesophagostomum dentatum* helminths but observed no effect on reducing parasitic infection. However, in a subsequent study, feeding a supplement (2%) consisting of a blend of *Saccharina latissima* (6%), *Ascophyllum nodosum* (6%) and fermented rapeseed (88%) tended to reduce parasitic fecal egg counts and the incidence of *A. suum* infection [104].

### 10.4. Meat Quality

The many bioactive compounds and phytochemicals present in seaweed make it an ideal potential animal feed supplement to improve meat quality, safety, and shelf life. Dietary macroalgae and extracts can increase omega-3 FA content, and contain antioxidants (i.e., carotenoids, chlorophylls, and polyphenols) that can increase antioxidant capacity in meat to enhance its nutritional value, meat color, sensory appeal, and extend shelf life by reducing lipid and protein oxidation and myoglobin degradation during storage [136]. Furthermore, some bioactive compounds in seaweed have potent antimicrobial activities that can reduce pathogenic bacteria such as *Salmonella*, *Escherichia coli*, and *Campylobacter* to reduce meat safety risks [136]. However, only a limited number of studies have been conducted to evaluate these potential benefits from feeding seaweed to growing-finishing pigs on carcass characteristics, meat quality, and food safety.

No effect of feeding extracts from *Ascophyllum nodosum* [288] and *Gracilaria lemaneiformis* [338] was observed for carcass characteristics (backfat, loin muscle depth or area) and carcass and lean meat yield. Some studies observed no effect on meat quality traits such as color and pH [135,318,319,339,340,341], while others reported slight changes in color [338,342] and reduced water absorption and drip loss [342]. A few studies showed improved shelf-life resulting from reduced lipid oxidation [318,319,320], improved oxidative stability [135,340], and reduced bacterial growth [320]. However, Moroney et al. [318] reported no effect of feeding a seaweed supplemented diet on microbial counts on fresh pork. Variable responses have been reported regarding changes in the nutritional profile of fresh pork from feeding seaweed diets to pigs including increased ash and cholesterol content [339], less fat and more protein [270], reduced omega-6:omega-3 [340], increased intramuscular fat in longissimus dorsi and altered amino acid and FA profiles [338]. Several studies have shown that seaweed supplemented diets increased iodine content of edible tissues [295,340,341,343], while Jerez-Timaure et al. [339] reported that feeding 4% *Macrocystis pyrifera* resulted in a reduction in manganese, iron, and copper in meat.

### 10.5. Summary of Responses from Feeding Various Seaweed Species, Extracts, and Blends

As previously described, because of the wide variation in nutritional and bioactive composition among numerous types of brown, green, and red seaweed species, it is useful to summarize our current knowledge of various biological responses that have been observed when feeding intact seaweed or extracts to swine for several reasons. First, some seaweed species may not be readily available in certain geographies but comparable alternatives with similar composition may provide similar benefits. Second, initial studies evaluating the potential benefits of feeding some seaweed species and commercial extracts have shown positive results, but more studies are needed to validate these responses for widespread commercial application. Finally, using the references for chemical composition data presented previously, prospective seaweed species with unique or high concentrations of bioactive compounds can be identified for future research studies.

Makkar et al. [108] indicated that the most common seaweed species used in livestock and poultry feed are the brown algae (*Phaeophyceae*) species of *Ascophyllum*, *Laminaria*, *Macrocystis*, *Nereocystis*, *Saccharina*, *Sargassum*, and *Undaria*, green algae (*Chlorophyceae*) species of *Ulva*, *Codium*, *Chaetomorpha*, and *Cladophora*, red algae species of *Pyropia*, *Porphyra*, *Chondrus*, *Gracilaria*, and *Palmaria*. As summarized in Table 8, Table 9, Table 10 and Table 11, the majority of studies that evaluated the use of seaweed in swine diets involved brown seaweed species, with intact *Laminaria sp*. and extracts (*n* = 44) followed by intact *Ascophyllum nodosum* and extracts (*n* = 15) representing most studies. Only 10 studies have evaluated green seaweed species of *Enteromorpha* sp. and *Ulva* sp., and only 9 studies evaluated potential swine performance and health benefits of red seaweed species *Chondrus crispus*, *Gracilaria lemaneiformis*, *Lithothamnium calcareum*, *Palmaria palmata*, and *Phymatolithon calcareum*. In contrast, a total of 25 studies evaluated various commercial extracts, products and blends.

## 11. Effects of Feeding Seaweed and Extracts to Poultry

Seaweed, its derivatives, and blends with other ingredients are also very useful sources of nutrients and feed additives containing bioactive components that provide antimicrobial, antioxidant, and anti-inflammatory properties to enhance health and productivity, and PUFAs, polyphenols, and pigments that enhance the quality and nutritional value of meat and eggs [137]. Previous studies have evaluated species from 5 genera of green seaweed (*Ulva*, *Enteromorpha*, *Codium*, *Cladophora*, *Chaetomorpha*), 7 genera of red seaweed (*Gracilaria*, *Chondrus*, *Gigertina*, *Kappaphycus*, *Palmaria*, *Polysiphonia*, *Sarcodiotheca*) and 11 genera of brown seaweed (*Sargassum*, *Fucus*, *Laminaria*, *Ascophyllum*, *Bifurcaria*, *Cystoseira*, *Hizikia*, *Macrocystis*, *Pelvetia*, *Saccharina*, *Undaria*) in poultry diets [137]. As previously discussed, the nutritional and chemical composition of seaweed is highly variable between species, geographic region, season, habitat, maturity, time of harvest, water temperature, and environmental and physiological variation which make it challenging to use accurate nutrient and bioactive compound concentrations when determining optimal inclusion rates during feed formulation [137]. Seaweed has also been evaluated as a pellet binder in poultry diets. El-Deek and Brikaa [363] evaluated the addition of a red seaweed (*Polysiphonia* sp.) added as a pellet binder up to 3% of duck diets and observed no effects on growth performance and carcass traits but some improvement in pellet hardness. Subakir et al. [364] evaluated the addition of up to 10% *Kappaphycus alvarzeii* and *Sargassum polycystum* as pellet binders in broiler diets and observed optimal pellet quality and growth performance when dietary levels of 2% or 5% were added, but there were no effects on carcass traits.

### 11.1. Broilers

Several reviews have been conducted to summarize the effects of feeding seaweed to poultry [137,140,365,366]. Andri et al. [367] conducted a systematic review and meta-analysis of the effects of feeding seaweed to broilers on growth performance which only included nine comparison from six studies four different seaweed species and a seaweed blend and reported no effect on FI but improved ADG and G:F. A more recent and comprehensive meta-analysis study for broilers was conducted by Yousaf et al. [368] that included 34 studies with 159 observations and showed significant improvements in body weight gain and feed conversion, with the greatest responses for Ross 308 and Arbor Acres genetic lines, and from feeding *Kappaphycus alvarezii* and *Chondrus crispus* species due to their high concentrations of polysaccharides and polyphenols that were associated with improving gut microbiome diversity and immunity.

Despite the many benefits that numerous seaweed species provide to enhance the health, productivity of food producing animals and the quality of food products they produce, they also present challenges when attempting to develop widespread use of seaweed and derivatives in poultry diets. Access to adequate supplies to seaweed is a major challenge in most countries around the world because poultry production occurs at substantial transport distances from the oceans where seaweed is produced and harvested [138]. Although several studies have shown multiple benefits of feeding various types of intact seaweed, seaweed extracts, and commercial blends and products to broilers, layers, ducks, geese, turkeys, and quail, some reports indicate no effects or negative outcomes. Some of the negative outcomes are due to poor experimental design, selecting the wrong seaweed species to achieve the desired response outcomes, and poor characterization of the chemical composition of the seaweed species evaluated due to the high variability in nutrient and chemical composition within and among species.

Seaweed also contains variable but generally high concentrations of dietary fiber (25–75% on a DM basis) which is comprised of many types of non-starch polysaccharides. These high concentrations of structural polysaccharides are found in the cell wall matrix of seaweed which enables them to survive harsh environmental sea conditions [120]. However, the rigidity of the cell wall matrix restricts the release of bioactive compounds [369] and limits nutrient digestibility [370]. These structural polysaccharides are a major challenge for poultry digestion [371]. Therefore, the need for using effective pre-treatment methods such as bacterial and fungal fermentation and the addition of appropriate carbohydrase enzymes limit the use of seaweed in resource-poor countries that do not have access or can afford these technologies [138]. Using bioprocessing techniques to extract valuable bioactive compounds can help capture the value of seaweed uses in poultry diets, but it is expensive, time consuming, and requires specialized equipment and processes to preserve biological activity [372,373].

Some compounds naturally present in seaweed affect the aroma, taste, and consumer acceptance of foods derived from poultry fed diets containing seaweed [374]. Seaweed sequesters heavy metals which cause animal health and human food safety concerns, especially because of increased marine water pollution, but some research has been initiated [375] using microorganisms to transform heavy metals into less toxic forms. Strict coastal regulation policies to minimize the accumulation of heavy metals are also needed [376]. A summary of nutritional and biological responses from feeding various seaweed species, blends and commercial products to broilers is provided in Table 12.

### 11.2. Layers

A summary of production, egg quality, and health responses from feeding various types of intact seaweed, extracts, and commercial blends is provided in Table 13. Depending on seaweed species evaluated and diet inclusion rate, several studies have shown improvements in egg production [415,416,417,418,419,420,421], while others reported reductions in egg production [422] or no change [415,423,424,425]. Similarly, variable responses were observed among studies for various measures of egg quality. However, several studies reported that diets containing seaweed supplements were effective in controlling a *Salmonella Enteritidis* challenge [425,426,427].

### 11.3. Other Poultry

#### 11.3.1. Ducks

An initial study evaluated feeding diets containing 1.5% or 3% *Polysiphonis* spp. to growing ducks and showed no differences in growth performance and carcass characteristics [445]. A subsequent study evaluated feeding increasing levels of *Polysiphonis* spp. in starter and finisher diets for commercial ducks and showed that up to 12% of this red seaweed can be used effectively in mash or pelleted starter diets, and up to 15% in finisher diets, without affecting growth performance and carcass quality [363]. Feeding diets containing a blend of moringa leaf powder (3%) and seaweed powder (3% *Gracilaria parvispora*) to Nageswari ducks (native to Bangladesh) resulted in significant improvements in growth performance, carcass yield and quality, and reproductive performance while maintaining normal physiological measures [417]. Muscovy ducks fed diets containing seaweed polysaccharides or seaweed enzymatic hydrolysate derived from a seaweed blend of 29% *Pyropia*, 24% *Palmaria palmata*, and 47% kelp for 28 days. Results showed that feeding either additive reduced FI and feed to gain ratio, improved intestinal morphology, altering intestinal microbiota, enhancing antioxidant capacity and immune function, and improving several metabolic measures [423]. Feeding diets containing 12.5% *Gracilaria* sp. waste and multi-enzyme supplement to ducks improved the blood lipid profile by decreasing serum cholesterol, triglycerides, low-density lipoprotein and increased high-density lipoprotein [424].

#### 11.3.2. Geese

One study has been conducted to evaluate the effects of feeding Zi geese diets containing 3% *Enteromorpha* powder [418]. Results showed that geese fed seaweed supplemented diet had improved egg production, laying rate, average daily egg weight, and egg yolk color, reduced FI and feed conversion ratio, and improved antioxidant status compared with geese fed the unsupplemented diet.

#### 11.3.3. Turkeys

Similar to geese, only one study has been conducted to evaluate the effects of feeding seaweed to turkeys. Abdel-Ghany et al. [419] fed diets supplemented with 1% or 2% *Ascophyllum nodosum* to Broad Breasted Bronze commercial turkeys from 10 weeks of age until market at 21 weeks of age. Feeding the seaweed supplemented diets improved ADG, but decreased FI, resulting in an improvement in G:F, but there was a reduction in carcass weight and abdominal fat, with no effects on plasma measures and meat quality except for changes in muscle color.

#### 11.3.4. Japanese Quail

Several studies have been conducted to evaluate the effects of feeding intact and fermented species of seaweed in Japanese and Jumbo quail diets on growth performance, carcass composition, meat quality, laying performance, egg quality, and various physiological and health measures. Supplementing Japanese quail diets with 1% or 2% *Sargassum siliquastrum* had no effect on ADG, FI, and final body weight, but improved G:F, nutrient digestibility, intestinal morphology, cecal microbial profile, antioxidant capacity, and immune response [425]. Similarly, feeding diets containing 3% *Chaetomorpha antennina*, *Sargassum wightii*, or *Gracilaria corticata* for 6 weeks had no effect on ADG and G:F compared with quail fed unsupplemented diets [426]. Adding up to 8% *Ulva* sp. to Jumbo qual diets had no linear or quadratics effects on FI, ADG, hematology measures, internal organs, carcass composition, and meat quality characteristics, but linearly decreased G:F and slaughter weights [420]. However, Hafsa et al. [421] used *Trichoderma reesei* in a solid-state fungal fermentation process to degrade the recalcitrant polysaccharides in *Ulva lactuca* before adding to growing Japanese quail diets at 1% or 2% and feeding unfermented and fermented seaweed diets for 42 days. Birds fed the fermented seaweed diets had improved ADG and G:F, greater dietary protein and fiber digestibility, increased cecal *Lactobacillus* and decreased cecal *Clostridium perfringens* and *Escherichia coli* counts, and greater antioxidant capacity. These results indicate that feeding diets containing up to 2% fermented *Ulva lactuca* promotes health and growth performance of quail. Diet inclusion of 6% to 9% *Gracilaria birdiae* is effective in mitigating the adverse health and performance effects cause by heat stress in Japanese quail [422]. Targhi et al. [446] reported that diet inclusion of up to 5% *Gracilariopsis persica* resulted in no effects on growth performance but enhanced immune function, improved meat quality, and increased the omega-3 FA concentrations in meat.

Male and female Japanese quail were fed diets with or without 1.5% or 3% *Ulva fasciata* or *Sargassum cinereum* for 14 weeks to evaluate effects on laying performance, egg quality blood lipid profile, and antioxidant status [427]. The was no effect on FI, but feeding seaweed supplemented diets improved egg laying rate, egg production, weight, and mass, and feed efficiency. In addition, seaweed supplementation improved eggshell thickness and egg yolk weight, index and color; decreased Haugh unit, total lipids, and cholesterol content of egg yolk; and improved fertility and hatchability percentages and blood lipid profile compared with quail fed unsupplemented diets. Similarly, serum and egg yolk cholesterol concentrations and susceptibility of egg yolks to oxidation were decreased by feeding diets containing up to 5% *Gracilariopsis persica* to 5-week-old laying quail for 12 weeks [447]. Laying quail diets supplemented with 20 or 40 mg/L of phycocyanin or fucoidan extracts resulted in improvements in egg production, egg mass, feed conversion, intestinal morphology and microflora composition, decreased egg yolk cholesterol and oxidation, but had no effects on egg weight and FI [448]. Results from a similar study showed that feeding these algal derivatives improved egg production, egg mass, fertility, and hatchability, but had no effect on immune responses [449].

In summary, results from multiple studies have shown significant benefits of adding several seaweed species to broiler, layer, duck, and quail diets to improve growth performance and egg laying rate, meat and egg quality, fertility and egg hatchability. Many of these beneficial responses appear to be associated with dietary seaweed supplementation through improved intestinal morphology, immune function, antioxidant capacity, and modulating the intestinal microbiome. Although feeding intact seaweed and seaweed extracts and blends have been shown to be effective dietary supplements, greater potency at lower diet inclusion rates can be achieved by feeding seaweed extracts and derivatives, perhaps with the exception of polysaccharides. Although polysaccharides function as prebiotics in some seaweed species, a few studies showed benefits of using fermentation to break down recalcitrant dietary fiber and improve feeding value. This is important because polysaccharides can bind nutrients and inhibit their digestion and absorption in the intestine [450]. Seaweed also contains high salt concentrations which can lead to wet litter, even when added at relatively low diet inclusion rates. Many species and sources of seaweed contain significant concentrations of heavy metals which may be detrimental to bird health and performance and potentially lead to food safety concerns for the meat and eggs produced.

## 12. Food and Feed Safety Hazards in Seaweed

However, the detoxifying capabilities of seaweed also lead to the bioaccumulation of several toxic substances that are food and feed safety liabilities because of their detrimental effects on human and animal health. Guidelines and regulations vary among types of toxic substances from being nonexistent to being widely variable among countries. Several studies have shown that seaweed is effective in removing pollutants such as industrial dyes, nitrogen, phosphorus, heavy metals and phenolic compounds from wastewater in simulated studies, but limited research has been conducted to determine seaweed’s effectiveness in real wastewater [451]. Heavy metals (i.e., aluminum, arsenic, cadmium, mercury, nickel, and lead) are commonly found in all seaweed species, but concentrations are highly variable among species and geographical location, and some can exceed international limits for food safety [113,452,453,454]. Seaweed also accumulates persistent organic pollutants (polycyclic aromatic hydrocarbons, PAH; polychlorinated biphenyls, PCBs; polychlorinated-dibenzo-p-dioxins, PCDDs; polychlorinated-dibenzofurans, PCDFs; polybrominated diphenyl ethers, PBDEs; and organic pesticides such as DDTs [453,455,456,457,458]. Some pathogenic microorganisms such as *Vibrio* spp., Shigatoxigenic *Escherichia coli*, *Listeria monocytogenes*, and *Salmonella* spp. were shown to survive at storage temperatures up to 22 °C on seaweed during storage [459], but the potential role of seaweed in the development of antimicrobial resistance and spread of antimicrobial resistant genes is poorly understood [460].

Biotoxins are naturally occurring toxic metabolites produced by cyanobacteria and dinoflagellates that grow in seaweed [461] and include palytoxin and domoic acid along with its analogues ciguatoxins and cyclin imines [462]. The dinoflagellate *Ostreopsis* sp. produces palytoxin in brown and red seaweed [463,464], and *Gambierdiscus* spp. produces ciguatoxins in all types of seaweed [465]. *Palmaria palmata* has been reported to produce kainic acid, which is a neuroactive toxin similar to domoic acid [114,466]. Prostaglandins, polycavernoside, aplysiatoxin, and debromoaplysiatoxin are other biotoxins produced by seaweed that have been linked to foodborne illnesses and deaths [467]. No regulations for biotoxins in seaweed have been established.

Allergic reactions have been observed for some red seaweeds (e.g., *Porphyrae tenera* and *Porphyra yezoenis*) that contain porphyrin that can cause hypersensitivity reactions [468], and allergenicity in *Ulva* spp. of green seaweed [469]. In contrast, several studies have shown anti-allergenic properties of brown seaweed [470,471,472]. There have been limited studies resulting in no regulations for allergens in food and feed [473].

Global plastic pollution has become a serious One Health crisis with detrimental health effects on humans, plants, animals, and ecosystems [474]. Because of their widespread distribution and accumulation in the marine environment, several studies have shown that macroalgae are capable of bioaccumulation of micro- and nanoplastics [475,476,477]. However, seaweed has the potential to be used to produce biodegradable alternatives to petrochemical-based plastics [474].

In addition, per- and polyfluoroalkyl substances (PFAS) are a class of synthetic chemicals used in numerous industrial and consumer products and applications, are widely distributed in the environment, are extremely resistant to degradation, and have also become a major One Health crisis affecting plant, animal, human, and ecosystem health [478]. Several studies have shown that seaweed is capable of bioaccumulating PFAS, and high concentrations can be found in heavily polluted areas resulting from wastewater discharge [479,480,481,482,483].

Radioactive nuclear waste discharged into the marine environment is also a health concern because of various radionuclides (e.g., cesium, plutonium, polonium, radium, uranium). Some studies have shown that seaweed is a bioaccumulator of radionuclides, and the impacts on these pollutants in the marine environment on climate change are largely unknown [484,485,486].

Because marine environments have become a concentrated source of all of these types of pollutants, and due to the ability of seaweed to detoxify ocean water, macroalgae have enormous benefits for improving marine ecosystem health, but robust food and feed safety monitoring systems and guidelines are needed to ensure that source being used in human foods and animal feeds pose no health threat. A summary of the relative hazard severity and links to animal feed and food for known potential contaminants of seaweed are shown in Table 14. Note that the heavy metals arsenic and cadmium, the micro mineral iodine, and the bacterial pathogen *Salmonella* rank the highest among all potential contaminants for concern and severity.

## 13. Assessing the Environmental and One Health Impacts of Seaweed and Extracts in Diets for Food Producing Animals

### 13.1. Environmental Impacts

Animal feed and manure are the main drivers of the environmental footprint of food animal production systems [487]. Data on the LCA of environmental impacts of feed ingredients are being compiled in the Global Feed LCA Institute (https://globalfeedlca.org/gfli-database/ accessed on 21 April 2026) for use as constraints in multi-objective feed formulation to develop nutritionally adequate, low environmental impact livestock and poultry diets [488,489,490]. The goal is to use this as a standardized approach for reducing the environmental footprint of food animal production systems and the meat, milk, and eggs that are produced. To accomplish this, LCA environmental impact data for multiple measures (e.g., global warming potential, land use, water use) and geographic locations where they are produced are needed for all major feed ingredients used in animal feed. There are limited LCA data for the various types of seaweed used in animal feeds, and because of the wide range in cultivated production, harvesting (wild vs. cultivated), and processing among various seaweed types grown in diverse geographic locations around the world, estimates are expected to be highly variable and specific to type and origin of seaweed. Furthermore, if LCA data were available for various intact seaweed sources, it would likely have limited contributions to low environmental impact diets when used as a nutrient source because it is generally limited to <5% of the total diet. Although LCA data can be determined for the production of seaweed extracts as feed additives to improve health, productivity, or reduce enteric methane emissions in ruminants, the uncertainty of the occurrence and magnitude of positive responses that can be measured in animal performance limits the usefulness of LCA data for seaweed extracts in this context.

Seaweed LCA studies have been conducted to assess the environmental impacts of seaweed cultivation, harvesting, and processing for use in bioenergy, food, feed, fertilizer, biomolecules, bioplastics, biostimulants, nanomaterials, construction materials, and cosmetic applications, and have resulted in a wide range in LCA estimates among studies due to differences in functional units, system boundaries, allocation methods, impact assessment methods, production conditions, and yield [4]. The limited number of LCA studies, along with variation in how system boundaries were defined and how data were represented and aggregated among studies, resulted in large variation in LCA estimates across cultivation and harvest systems, even within the same classification. The majority of LCA determinations have been conducted for offshore cultivation and harvest systems followed by harvest of wild and beach cast seaweed, with very few studies involving land-based, near-shore, and IMTA cultivation and harvest systems [35]. Because of the large variation in LCA methods used to estimate global warming potential among studies, no clear ranking or comparisons could be made. However, several recommendations have been suggested to reduce the environmental impact of seaweed production and biorefining systems including adopting renewable energy sources, use biodegradable materials for cultivation infrastructure, minimize distance between cultivation and processing sites, optimize biomass productivity and composition, implement seaweed polyculture systems, and align harvest strategy with market flexibility [35].

Although results from LCA studies have shown that seaweed can provide climate change and other environmental benefits, there is not enough robust scientific evidence to indicate net negative carbon emissions across the entire supply chain that is necessary to support seaweed as a climate solution because environmental impacts vary widely based on cultivation system, seaweed species and products, and fate in various markets [491]. There are many knowledge gaps that need to be addressed to accurately quantify climate benefits of seaweed that include using whole life cycles over time [491]. Environmental hotspots in cultivation and harvesting and challenges for future design have been described in many LCA studies and reviews [4,35,492,493,494,495]. The drying process consumes a large amount of energy and is the main contributor to the climate impact of seaweed processing [492,496]. As previously discussed, processing methods not only affect environmental impacts, but they can also alter nutritional and phytochemical composition which can affect their nutraceutical and anti-methanogenic effectiveness [497,498].

Only a few studies have been conducted to apply LCA methodology to seaweed use in animal feed. Ridoutt et al. [499] used secondary data from the literature to model GHG emissions from adding *Asparagopsis taxiformis* to beef cattle diets only during the feedlot phase of production but did not follow ISO-compliant LCA methods and did not account for additional carbon dioxide and nitrous oxide emissions associated with land use, feed production, and manure management. Nilsson et al. [492] determined that the climate change impact of wet *Saccharina latissima* in Ireland and showed that the drying process is a primary contributor to the estimated GHG emissions from producing dried seaweed for feed and food use. Méité et al. [500] reported that the combination of feeding high dietary levels of *Asparagopsis* to dairy cows, using a cow toilet system to separate urine and feces, and acidification of slurry with sulfuric acid were most effective in reducing global warming potential, eutrophication and acidification potential, and abiotic depletion on commercial dairy farms but all three practices involved trade-offs. Thomas et al. [7] evaluated the broader life cycle impacts of using diverse seaweeds and processing methods in multiple ruminant production systems and reported that while some seaweed species can reduce enteric methane production in ruminants at the farm level in the short-term, when upstream energy-intensive harvesting, drying, processing and long transport distances were included in LCA assessments, the benefits are greatly diminished. In addition, moderate methane reductions contributed to only marginal climate change impacts when carbon dioxide and nitrous oxide emissions were included. Little is known about the effects of feeding seaweed and extracts on nitrous oxide emissions, which is a more potent GHG than methane, or its impacts on GHG emissions from swine and poultry. However, results from one study has shown that phlorotannins in *Himanthalia elongata* alter protein digestion by diverting more nitrogen toward feces resulting in a 40 to 50% reduction in ammonia emissions from manure of dairy cows [193]. More investigations are needed to explore this potential benefit of dietary seaweed for reducing nitrogen losses from livestock and poultry production systems.

### 13.2. One Health Tradeoffs for Using Seaweed in Livestock and Poultry Diets

Macroalgae biomass production has several environmental sustainability advantages compared with terrestrial biomass production because it does not require use of arable land, freshwater, and synthetic fertilizer, and therefore, does not compete with these natural resources for animal feed and food production [3,4,5,6,7]. In addition, this enormous global seaweed biomass sequesters carbon, reduces eutrophication by absorbing nitrogen and phosphorus and other nutrients, and detoxifies oceans by absorbing heavy metals, microplastics, PFAS, and persistent organic pollutants, which helps protect the health of diverse marine ecosystems, but the bioaccumulation of these contaminants also causes food and feed safety concerns [462]. Therefore, the potential feed and food safety risks must be accurately assessed relative to the environmental benefits of feeding seaweed and seaweed extracts to food producing animals.

Numerous seaweed species contain substantial amounts of bioactive compounds that can also have a substantial indirect benefit on reducing the environmental footprint of livestock and poultry production by improving animal health and productivity while reducing mortality. However, although a commercially produced bromochloromethane has been shown to be effective in reducing 93% of enteric methane emissions in beef cattle [501], it is also classified as a potential human carcinogen, is regulated in drinking water, and is an ozone-depleting substance that is banned from commercial use in several countries [502]. Because of the chemical structure similarity between bromochloromethane and bromoform, and bromoform emissions from seaweed have been identified as a significant contributor to atmospheric halocarbon fluxes [503] associated with ozone depletion, its impacts on the environment and animal health are a regulatory concern. There are multiple tradeoffs between positive and negative environmental impacts, animal health benefits and risk factors, and improvements in quality of meat, milk, and eggs versus potential food safety concerns that are associated with the use of seaweed and bioactive compounds in animal diets. As a result, it would be useful to develop a risk/benefit assessment evaluation framework to quantitatively characterize the value that seaweed can provide in global livestock and poultry production systems for the efficient production of safe, nutritious, and high-quality meat milk and eggs with a lower environmental footprint compared with conventional food animal production systems.

## 14. Strategies to Increase Seaweed Use in Animal Feed

Several barriers must be overcome for seaweed to become a viable resource with multiple uses in the global feed and livestock industry, including reducing cost and emissions associated with cultivation and harvesting, consistency of frequency and quantity of supply, cost and effectiveness of drying for long-term storage, and creating bioprocessing infrastructure in close proximity to cultivation sites. Pantis et al. [504] conducted a comprehensive SWOT (Strengths, Weaknesses, Opportunities, Threats) analysis of the environmental impacts of seaweed production and harvest. The strengths included fast biomass accumulation without requiring arable land and fresh water, diverse nutrient and phytochemical composition, and natural sequestration of carbon, nitrogen, and phosphorus. Weaknesses include insufficient, inconsistent, or outdated inventory data for LCAs, high energy demands for processing and drying fresh biomass along with complex downstream extraction requirements, and highly variable chemical composition among species, seasons, and location. However, considerable opportunities exist for growing demand and emerging markets, integration in blude and circular bioeconomy frameworks, and multi-product extraction technologies that maximize recovery of high value compounds. The biggest threats are scaling barriers, environmental toxicity impacts, and economic viability.

The future of using seaweed and its derivatives in animal feed involves a series of tradeoffs that must be reconciled.

1. Connect the key stakeholders in the supply chain—it is imperative that communication, technology sharing, and business linkages and networks be developed between seaweed harvesters and producers, bioprocessors, animal nutritionists, feed industry practitioners, and livestock and poultry producers to explore economical and environmentally sustainable ways of increasing seaweed supply to match an increasing application and demand for its use in animal feed. This includes locating dehydration and bioprocessing facilities in close proximity to production and harvest locations to minimize transport cost and preserve quality.
2. Develop an international open access chemical composition database—numerous published studies and global organizational websites provide chemical composition data on numerous seaweed species but there is no open access international database of the nutrient, phytochemical, and hazardous compound concentrations for seaweed. Creating a dynamic comprehensive database would provide multiple benefits for increasing the use of seaweed and its extractives in animal feed that include new potential high value bioactive sources, assess relative nutritional suitability for optimizing feeding applications for specific animal species, serve as a general, comparative baseline with chemical composition of terrestrially produced feed ingredients, and identify locations where the presence and concentrations of hazardous compounds may be a feed and food safety concern.
3. Standardize analytical methodologies and chemical composition reporting—sample grinding, analysis, and metrics used to report chemical composition are more challenging than those used for terrestrial feed ingredients and can lead to inaccurate assessment of the true nutritional value. The highly complex cellular structure, recalcitrant fiber interactions with nutrients, relatively high proportion of non-protein nitrogen, and unique biochemical properties of seaweed require use of consistent and specialized analytical and measurement techniques.
4. Use “green” and non-destructive bioprocessing technologies to maximize yields and compound purity, preserve phytochemical potency and bioactivity, and minimize environmental footprint of extraction processes to produce concentrated bioactive feed additives.
5. Develop and implement commercial scale solid state fungal or bacterial fermentation technologies to break down fiber and anti-nutritional factors, and enhance amino acid concentrations, lipid profiles, and mineral digestibility to increase diet inclusion rates and use in swine and poultry diets.
6. Develop enhanced bromoform and associated phytochemical extraction techniques to optimize yield, composition, and bioactivity as an effective dietary enteric methane mitigation strategy in ruminants.
7. Conduct essential scientific studies to evaluate feed and food safety, and physiological response claims of intact seaweed and extracts to obtain regulatory approval for use in animal feed.
8. Conduct life cycle assessments of intact seaweed and extracts used in animal feed and production systems and evaluate environmental and economic tradeoffs compared with common feed ingredients, by-products, and feed additives.
9. Develop a risk assessment evaluation framework to quantitatively characterize the value that seaweed can provide in global livestock and poultry production systems for the efficient production of safe, nutritious, and high-quality meat milk and eggs with a lower environmental footprint compared with conventional food animal production systems.

## 15. Conclusions

Seaweed has served as a valuable and sustainable animal feed resource for small scale, extensive livestock and poultry farms for centuries, but it is an underutilized as a nutrient and bioactive compound source in animal feeds in medium- to large-scale intensive livestock and poultry production systems globally. The declining availability of land and freshwater, and reliance on synthetic fertilizer to produce crops used in animal feed have led to the need to explore abundant supplies of seaweed biomass as a nutrient and bioactive compound resource in animal feed. However, the future of using seaweed and its derivatives in animal feed involves multiple tradeoffs that must be reconciled.

There are multiple tradeoffs between positive and negative environmental impacts, animal health benefits and risk factors, and improvements in quality of meat, milk, and eggs versus potential food safety concerns that are associated with the use of seaweed and its bioactive compounds in animal diets. Although seaweed production has many environmental benefits in a marine environment, capturing and quantifying these benefits as a source of nutrients and bioactive compounds in animal feed and production systems is challenging because of low diet inclusion rates and inconsistent productivity, health, and food product quality responses. Bromoform present in some seaweed species can reduce enteric methane production when fed to ruminants at the farm level in the short-term, but when the upstream energy-intensive harvesting, drying, processing and long transport distances, the benefits are greatly diminished. Seaweed is very effective in detoxifying oceans by absorbing heavy metals, microplastics, PFAS, and persistent organic pollutants, which helps protect the health of diverse marine ecosystems, but the bioaccumulation of these contaminants also causes food and feed safety concerns relative to human and animal health. However, many bioactive compounds present in seaweed improve the nutritional profiles and healthfulness of meat, milk, and eggs for human consumption, and several phytochemicals present in seaweed provide antimicrobial, antiviral, antioxidant, and immune system benefits to improve animal health. Therefore, a risk/benefit evaluation framework is needed to characterize the environmental sustainability and One Health benefits and limitations of feeding seaweed and seaweed extracts to food producing animals.

## Figures and Tables

**Table 1:** Countries, continents and the main seaweed species cultivated in 2018 (adapted from [26,27,28]).

Country/Continent (% of Global Total)	Major Seaweed Species
China (56.8%)	*Laminaria japonica*
	*Gracilaria* spp.
	*Porphyra* spp.
Indonesia (26.8%)	*Eucheuma* spp.
	*Gracilaria* spp.
South Korea (5.1%)	*Laminaria japonica*
	*Porphyra tenera*
	*Undaria pinnatifida*
Philippines (4.2%)	*Kappaphycus alvarezii*
North Korea (1.6%)	*Laminaria japonica*
Japan (1.2%)	*Porphyra tenera*
	*Undaria pinnatifida*
	*Laminaria japonica*
Malaysia (0.53%)	*Kappaphycus alvarezii*
North America (1.4%)	*Saccharina latissima*
	*Nereocystic luetkeana*
	*Macrocystis pyrifera*
	*Alaria esculenta*
	*Palmaria palmata*
	*Laminaria digitata*
Chile (0.3%)	*Porphyra* spp.
	*Saccharina latissima*
Europe (0.8%)	*Gracilaria gracilis*
	*Alaria esculenta*
	*Codium tomentosum*
	*Palmaria palmata*
	*Ulva* spp.
	*Kappaphycus alvarezii*
	*Eucheuma* spp.
	*Gracilaria* spp.
Africa (0.4%)	*Eucheuma* spp.
	*Kappaphycus* spp.
	*Gracilaria* spp.
	*Ulva* spp.

**Table 2:** Common uses of seaweed species produced and traded (adapted from [9]).

Seaweed Species	Human Food	Food Hydrocolloids	Parmaceuticals/Nutraceuticals	Animal/Aquatic Feed	Biopackaging/ Plastic Alternatives	Biofertilizer/Biostimulants	Biofuels	Waste Treatment
Brown seaweed								
*Fucus* spp.			X					
*Laminaria* spp.	X	X	X				X	
*Laminaria hyperborea*	X	X	X				X	
*Lessonia* spp.				X				
*Macrocystis pyrifera*			X					
*Saccharina japonica*	X	X	X		X			
*Saccarina latissima*	X						X	
*Sargassum* spp.				X		X		
*Undaria pinnatifida*	X		X					
Green seaweed								
*Caulerpa* spp.				X				
*Codium fragile* spp. *Californicum*	X							
*Ulva* spp.				X				X
Red seaweed								
*Asparagopsis taxiformis*	X			X				
*Chondracanthus chamissoi*	X	X						
*Chondrus crispus*	X							X
*Eucheuma* spp.	X	X		X				
*Eucheuma denticulatum*	X	X		X				
*Gelidium* spp.	X	X			X			
*Gigartina skottsbergii*		X	X					
*Gracilaria* spp.	X	X		X	X			X
*Gymnogongrus furcellatus*	X	X						
*Kappaphycus alvarezii*	X	X	X			X		
*Kappaphycus striatum*	X	X	X			X		
*Mazzaella laminarioides*	X	X						
*Palmaria palmata*	X							
*Pterocladia* spp.	X	X			X			
*Pyropia*/*Porphyra tenera*	X							
*Pyropia*/*Porphyra yezoensis*	X							
*Sarcothalia crispate*	X	X						

Common uses of each specific seaweed species listed are designated with an “X”.

**Table 3:** Summary of the advantages and disadvantages of methods used to extract bioactive compounds from seaweed (adapted from [78]).

Method	Advantages	Disadvantages
Conventional chemical extraction	Well-established methods	Long extraction time High energy and water use Precipitation and alcohol recovery are expensive Acids and bases can degrade polysaccharides Chemical solvents can adversely affect health
Supercritical fluid extraction	Uses non-toxic and non-flammable solvents Supercritical carbon dioxide can be easily removed from final extract No degradation and structural disruption of bioactive compounds	High acquisition cost High pressure required to maintain solvent in a critical state which can degrade bioactive compounds
Microwave assisted extraction	Short extraction time Requires small amounts of solvent Preserves product quality	Yield is reduced due to degradation of polysaccharide molecular structures
Enzyme assisted extraction	Relatively inexpensive Potential for high bioactive compound yield Gentle conditions preserve bioactivity in sensitive compounds	Extraction efficiency depends on enzyme characteristics Extraction yield depends on optimizing time, temperature, and pH conditions of enzymes Risk of degradation of target compounds by non-specific enzymes
Ultrasound assisted extraction	Relatively simple method Short extraction time Operation at low temperatures Low amounts of solvents required High yields of bioactive compounds	Degradation and structural modification of polysaccharides
Pressurized solvents extraction	High extraction efficiency using fewer solvents Relatively short extraction time	High cost of high-pressure equipment High temperature degrades thermolabile compounds

**Table 4:** Summary of the human and animal health benefits of various bioactive compounds in target seaweed species (adapted from [49,78]).

Health Benefit	Bioactive Compounds	Target Seaweed Species
Anti-coagulant	Polysaccharides (sulfated galactan, polycarboxyl ulvans, low molecular weight)	Brown—*Sargassum fusiforme* Green—*Udotea flabellum*, *Ulva lactuca* Red—*Gelidiella acerosa*
Anti-diabetic	Polysaccharides (fucoidan, sulfated pyruvylated polysaccharide)	Brown—*Sargassum pallidum*, *Sargassum wightii* Red—*Gracilaria edulis*
Anti-inflammatory	Polysaccharides (fucoidan, sulfated galactan) Fatty acids (eicosapentaenoic acid, EPA; saturated fatty acids, SFA; monounsaturated fatty acids, MUFA; PUFA; omega-3; omega-6) Pigments (fucoxanthin, chlorophyll a, pheophytin-α, β-carotene)	Brown—*Cystoseira crinite*, *Fucus spiralis*, *Saccharina japonica*, *Sargassum autumnale*, *Sargassum binderi*, *Sargassum confusum*, *Sargassum fusiformis*, *Sargassum fulvellum*, *Sargassum siliquastrum*, *Sargassum swartzii*, *Undaria pinnatifida*, Green—*Codium fragile*, *Ulva intestinalis*, *Ulva lactuca* Red—*Gracilaria gracilis*, *Palmaria palmata*
Anti-microbial	Terpenoids (diterpenoids, sesquiterpenoid) Pigments (fucoxanthin) Polyphenols (*p*-hydroxybenzoic, coumaric, and ferulic acids)	Brown—*Canistrocarpus cervicornis*, *Dictyota dichotoma*, *Gongolaria indica*, *Padina pavonica*, *Sargassum angustifolium*, *Stypopodium zonale* Green—*Ulva lactuca* Red—*Gracillaria dura*, *Kappapycus alvarezii*
Antioxidant	Polysaccharides (fucoidan, ulvan) Terpenoids Fatty acids (EPA, PUFA) Proteins and peptides Pigments (chlorophyll a and b, carotenoids, fucoxanthin, xanthophyll) Polyphenols (coumaric acid, ferulic acid, flavonoids, phlorotannins) Vitamins (A, B_1_, B_2_, B_3_, B_6_, B_9_, C, E)	Brown—*Cladostephus spongiosus*, *Dictyota cervicornis*, *Dictyota dichotoma*, *Ecklonia maxima*, *Fucus virsoides*, *Gongolaria barbata*, *Nizamuddinia zanardinii*, *Padina boryana*, *Padina pavonica*, *Phyllariopsis brevipes*, *Sargassum fulvellum*, *Sargassum fusiforme*, *Sargassum tenerrimum*, *Undaria pinnatifida*, *Sargassum wightii* Green—*Caulerma racemosa*, *Caulerpa chemnitzia*, *Cladophora vagabunda*, *Codium adhaerens*, *Ulva australis*, *Ulva intestinalis*, *Ulva lactuca* Red—*Acanthophora spicifera*, *Ceramium virgatum*, *Gigartina acicularis*, *Gracilaria corticate*, *Gracilariopsis lemaneiformis*, *Grateloupia turuturu*, *Hypnea musciformis*, *Laurencia tristicha*, *Odonthalia dentata*, *Palmaria palmata*, *Pyropia yezoensis*
Anti-protozoal	Polyphenols (ellagic acid)	Brown—*Padina boryana*
Anti-tumoral	Polysaccharides (fucoidan, sulfated galactans, porphyran, carrageenan) Terpenoids (diterpenes) Pigments (fucoxanthin) Polyphenols	Brown—*Dictyota bartayresiana*, *Fucus vesiculosus, Ecklonia radiata*, *Sargassum cinereum*, *Sargassum elegans*, *Sargassum pallidum*, *Sargassum tenerrimum*, *Undaria pinnatifida* Green—*Capsosiphon fulvescens*, *Codium isthmocladum*, *Udotea flabellum*, *Ulva conglobate*, *Ulva lactuca* Red—*Chondrus armatus*, *Pyropia haitanensis*
Anti-viral	Sulfated polysaccharides (fucoidan, carrageenan, laminarin, ulvan) Lectins Glycolipids Polyphenols (phlorotannins)	Brown—*Adenocystis utricularis*, *Dictyopteris jolyana*, *Dictyota bartayesiana*, *Dictyota mertensii*, *Ecklonia cava*, *Fucus evanescens*, *Fucus vesiculosus*, *Hydroclathrus clathratus*, *Kjellmaniella crassifolia*, *Laminaria abyssalis*, *Lobophora variegata*, *Padina gymnospora*, *Saccharina japonica*, *Sargassum filipendula*, *Sargassum fluitans*, *Sargassum henslowianum*, *Sargassum horneri*, *Sargassum mcclurei*, *Sargassum polycystum*, *Sargassum swartzii*, *Sargassum vulgare*, *Sargassum withtii*, *Spatoglossum schroederi*, *Stypopodium zonale*, *Turbinaria decurrens*, *Turbinaria ornata*, *Undaria pinnatifida*, *Zonaria tournefortii* Green—*Codimum fragile*, *Penicillus capitatus*, *Ulva lactuca* Red—*Alsidium seaforthii*, *Asparagopsis armata*, *Boergeseniella thuyoides*, *Chondrus crispus*, *Cryptonemia crenulate*, *Eucheuma denticulatum*, *Gelidum spinulosum*, *Gigartina skottsbergii*, *Gracilaria corticata*, *Gymnogongrus griffithsiae*, *Gymnogongrus torulosus*, *Halymenia floresii*, *Hypnea musciformis*, *Osmundaria obtusiloba*, *Plocamium cartilagineum*, *Pterocladia capillacea*, *Solieria chordalis*, *Solieria filiformis*, *Sphaerococcus coronopifolius*
Cardiovascular disease protection	Polysaccharides (fucoidan) Fatty acids (EPA, SFA, MUFA. PUFA) Proteins and peptides Pigments (fucoxanthin)	Brown—*Alaria esculenta*, *Fucus vesiculosus*, *Sargassum mcclurei*, *Sargassum wightii*, *Undaria pinnatifida* Green—*Ulva intestinalis*, *Ulva lactuca* Red—*Curdiea racovitzae*, *Gelidium spinosum*, *Gracilaria gracilis*, *Mazzaella japonica*, *Palmaria palmata*, *Porphyra dioica*, *Sphaerococcus coronopifolius*
Neurodegenerative disease protection	Polysaccharides (fucoidan) Fatty acids Pigments (fucoxanthin, carotenoids) Polyphenols (Phlorotannins)	Brown—*Eklonia cava*, *Ecklonia radiata*, *Sargassum fusiforme*, *Undaria pinnatifida*

**Table 5:** Summary of responses from studies evaluating dietary inclusion of selected seaweed species on nutritional and performance responses in lactating dairy cows.

Seaweed Species	Responses	References
Brown algae		
*Ascophyllum nodosum*	Increasing diet inclusion rates had quadratic effects on dry matter intake (DMI) and OM, acid detergent fiber (ADF), and neutral detergent fiber (NDF) digestibility. No effect on milk yield and milk fat, protein, lactose, and urea N conentrations. Other studies showed improved energy status, increased milk fat and iodine content, and reduced nutrient digestibility	[193,194,195,196,197,198]
*Fucus serratus*	No effect on milk fat and protein content and yield of fat, protein, lactose, and fat- and protein-corrected milk, feed efficiency, milk N efficiency, and somatic cell count	[199]
*Saccharina latissima*	Feeding 4% ensiled seaweed had no effects on methane production, DMI, and milk production; Other studies showed improved milk FA profiles but increased iodine and reduced calcium content in milk	[198,199,200,201]
*Sargassum johnstonii*	Feeding a combination of soybean straw and seaweed in total mixed rations (TMR) reduced DMI and milk yield	[202]
*Sargassum muticum*	Feeding 2% ensiled seaweed had no effects on DMI and milk production	[198]
*Sargassum wightii*	Seaweed supplementation increased milk yield and fat corrected milk yield but had no effect on nutrient intake and digestibility	[203]
Red algae		
*Asparagopsis armata*	Feeding seaweed steeped with canola oil had no effect or decreased DMI and milk yield and energy corrected milk, and bromoform concentrations in milk were safe for consumption	[204,205,206]
*Asparagopsis taxiformis*	Feeding 0.5% reduced methane emissions, altered VFA production, decreased DMI and milk yield but had no effect on milk composition except for decrease lactose yield; increasing dietary levels reduced feed intake (FI) and methane emissions with no change in bromoform concentration in milk; time spent eating and saliva production increased but no effect on rumination time	[207,208,209,210,211,212,213]
*Chondrus crispus*	No effect on milk fat and protein content, yield of fat, protein, lactose, and fat- and protein-corrected milk, feed efficiency, milk N efficiency, and somatic cell count; feeding 6% DMI had no effect on milk yield and composition but decreased methane production	[199,214]
Commerical extracts and seaweed blends		
Calcified seaweed + marine sourced MgO	Feeding marine-sourced rumen buffer to grazing lactating cows increased milk production, energy, fat, and fat corrected milk yield	[215]
Mixture of *Ascophyllum nodosum*, *Fucus vesiculosus*, and *Asparagopsis taxiformis*	No effect on milk yield but increased milk iodine and reduced milk urea concentrations	[216]
Commercial seaweed extract	Increased DM, OM, crude protein (CP), ether extract (EE), and nitrogen free extract (NFE) digestibility, increased total digestible nutrients (TDN) and digestible energy (DE) intake, rumen pH, total VFAs, and reduced rumen ammonia N concentrations; Other studies showed that increasing dietary inclusion had no effect on ADF and NDF digestibility, increased milk iodine concentrations, milk yield, and milk fat, lactose, solids non-fat, total solids, and ash content	[217]
Brown seaweed by-products	Feeding up to 4% seaweed by-products to lactating dairy cows in transition had no effect on DMI, weight gain, feed efficiency, milk yield and composition	[218]
Supplement mixture of *Ulva rigida*, (80%), *Sargasum muticum* (17.5%) and *Saccorhiza* polyschides (2.5%)	Supplement improved iodine and selenium and reduced molybdenum metabolic status but increased milk iodine and arsenic content which is a potential concern	[219]
Supplement mixture of 91% *Ascophyllum nodosum* and 9% *Laminaria digitata* (DM basis)	Feeding increasing levels had no effect on milk yield, feed efficiency, and energy corrected milk yield but milk protein, casein, selenium, and copper concentrations decreased while milk iodine and arsenic content increased	[220]
1:1:1 blend of *Kappaphycus alvarezii* powder, sap powder, and *Gracilaria salicornia* powder	Feeding up to 3% of diet had no effect on DMI, body weight, and feed efficiency	[221]

**Table 6:** Summary of responses from studies evaluating dietary inclusion of selected seaweed species on nutritional and performance responses in beef cattle.

Seaweed Species	Responses	References
Brown seaweed		
*Ascophyllum nodosum*	No effect on growth performance, NDF, ADF, and nutrient digestibility, rumen fermentation, variable responses on methane production and yield, but improved marbling scores and quality grades of beef carcasses	[223,224,225]
*Undaria pinnatifida*	Feeding 2% by-product improved growth performance, immune response, and FA profile in meat and reduced cholesterol content in meat	[226]
Red seaweed		
*Asparagopsis taxiformis*	Adding 0.10% and 0.20% reduced methane emissions by 40% and 98% and increased ADG by 53% and 42%, respectively, with no negative effects on FI, G:F, rumen function, or meat quality; inclusion of 0.25% and 0.5% reduced methane emissions by 45% and 68%, respectively, with no effects on ADG, carcass quality, strip loin chemical analysis and shear force, or consumer preference; methane emissions were reduced by 38% in grazing beef cattle without affecting DMI, ADG, and final body weight	[227,228,229]
*Mazzaella japonica*	Quadratic increase in DMI and linear decrease in DM digestibility with increasing diet inclusion rates in high forage diets but no effect on OM, CP, NDF, or starch digestibility, linear increase in N intake and fecal N but no effect on urinary N, rumen pH, total VFAs, and methane yield	[230]
Commercial extracts and blends		
Commercial extract from *Ascophyllum nodosum*	Application to endophyte infected tall fescue pasture had variable time effects on grazing cattle rectal temperature, improved monocyte immune cell response, and increased carcass marbling scores, but no effect on ADG; steers inoculated with *E. coli* O157:H7 had reduced fecal shedding, similar rumen pH and VFA concentrations; short-term diet supplementation before slaughter increased marbling scores and various meat quality and shelf-life attributes; no effect on DMI, ADG, G:F, digestibility, ruminal fermentation, and muscle FA profile but reduced methane production by 8%; increased antioxidant activity in endophyte infected forage and grazing cattle but variable effects on reducing respiration rate of heat stressed cattle and immune function	[225,231,232,233,234,235,236,237]
Canola oil infused with *Asparagopsis armata*	Feeding 25 mg/kg DMI had no effect on DMI but improved G:F, ADG, and final body weight, reduced methane production and yield, tended to increase hot carcass weight but had no effect on meat quality and no bromoform residues were detected in muscle, fat, and kidney	[238]
*Asparagopsis taxiformis* blends with canola oil	Feeding 17 to 51 mg/kg DMI reduced methane emissions by 64 to 99% with no effect on rumen temperature, pH, reduction potential, VFA and ammonia production, growth performance and carcass characteristics; no detectable levels of bromoform in carcasses and iodide and bromide concentration in kidneys were not a food safety concern	[239]
*Himanthalia elongata* extract	Increased NDF and ADF digestibility but had no effect on methane production or yield, ammonia, protozoa counts, total VFAs, and rumen pH	[224]
Rumen buffer from *Lithothamnium calcareum* extract	Feeding 0.5% calcified seaweed buffer had no effect on rumen fermentation and disappearance kinetics	[240]

**Table 8:** Summary of swine studies evaluating nutritional and biological responses from feeding brown seaweed species.

Brown Seaweed (*Phaeophyceae*)	Summary of Responses	References
*Ascophyllum nodosum*	No effect on growth performance, carcass characteristics, and intestinal morphology, improved antioxidant enzyme activity and fecal scores in weaned pigs; reduced *E. coli* and increased *Lactobacilli* in the small intestine; improved blood, liver, ileum lipid profiles; increased mineral digestibility, serum concentrations, bone mechanical strength, and iodine concentration in edible tissues	[150,284,288,295,299,344,345,346,347,348]
*Ascophyllum nodosum* extract	Sows fed extracts in low omega-6:omega-3 diets had reduced inflammatory severity and enhanced innate immunity, but offspring had variable growth performance responses and no effects on piglet oxidative status and immune response in weaned pigs challenged with *Salmonella typhimurium*; reduced pro-inflammatory response with lipopolysaccharide (LPS) challenge in colon	[271,272,273,323,349]
*Fucus serratus*	Phlorotannin extracts decreased *in vitro* nutrient digestibility indicating that diet inclusion rate should be based on phenolic concentration rather than whole seaweed inclusion rate	[345]
*Fucus serratus* extract	Decreased pro-inflammatory response with LPS challenge in colon	[349]
*Laminaria digitata* and extracts	Dietary inclusion of 300 ppm was optimal for reducing intestinal *Enterobacteriaceae* populations and increasing mucin and cytokine gene expression in LPS challenged pigs; feeding wet extract resulted in greater *Bifidobacterium* and *Lactobacillus* counts and prebiotic effects in the ileum than when feeding spray dried extracts to weaned pigs; 10% dietary inclusion improved multiple indicators of piglet health and stress reduction post-weaning without supplemental carbohydrases; increased oxidative stability, reduced SFA content and lipid oxidation, and increased bromine and iodine content of meat without affecting growth performance	[311,313,314,315,318,319,340,343,346,350]
*Laminaria hyperborea*	Greater reduction in *Bifidobacterium* sp. counts than *L. digitata*, and *Enterobacteriaceae* counts were reduced *in vitro*	[311]
*Laminaria japonica*	Diet inclusion up to 5% increased DM and ash digestibility, reduced OM and fiber digestibility, and increased FI, ADG, G:F, fecal amylase and lipase activity, and changed serum amino acid profile	[351,352]
*Laminaria* sp. extracts	Feeding laminarin and fucoidan extracts to sows reduced fecal shedding of *Salmonella typhimurium* and increased *Lactobacillus*, and improved gut morphology, immune response, immunoglobulin A (IgA) and immunoglobulin G (IgG) in colostrum and circulatory IgG in piglets, and enhanced immune function and colonic microflora in piglets at weaning; piglets had lower *E. coli* populations in cecum, increased villi height to crypt depth ratio, improved ADG and G:F, and had less diarrhea post-weaning; some studies showed no effect on growth performance in weaned and growing pigs but reductions in fecal and intestinal *Salmonella typhimurium*, reduced gene expression of proinflammatory cytokines in colonic tissue of challenged pigs, and improved intestinal health through changes in gastrointestinal microbiome and increased butyrate production; other studies showed that feeding laminarin and fucoidan extracts may alleviate the need for feeding high lactose diets to weaned pigs, improve N and gross energy digestibility, reduced fecal *E. coli* counts, and improve growth performance similar to feeding pharmacological levels of ZnO but by different mechanisms; improved gut health by reducing intestinal *Enterobacteria* sp. and increasing *Lactobacilli* sp. and VFAs in colon have been observed, and laminarin and fucoidan polysaccharides enhance meat quality and by increasing total antioxidant capacity and reducing lipid oxidation	[147,263,264,265,266,267,268,270,277,290,291,292,293,303,307,309,311,312,316,317,320,322,325,326,327,353]
*Ecklonia cava*	Improved ADG and DM, energy, and CP digestibility; increased cecal *Lactobacillus* sp. and decreased cecal *E. coli* and *Clostridium* sp. in weaned pigs	[298,310]
*Macrocystis pyrifera*	Had no effect on milk production and composition, litter weight gain, and weaning to estrus interval postweaning when fed to lactating sows, but feeding to finishing pigs increased ash content and reduced intensity of red color in meat	[278,339]
*Saccharina latissima*	Fermenting before feeding enhances gut health and immune function, and induces localized immunomodulatory effects and minor gut microbiota changes during intestinal helminth infection	[337,346,354]
*Undaria pinnatifida*	Enhanced immune response and intestinal microflora composition in growing pigs	[324]

**Table 9:** Summary of swine studies evaluating nutritional and biological responses from feeding green seaweed species.

Green Seaweed (*Chlorophyceae*)	Summary of Responses	References
*Enteromorpha* spp.	Enriching with zinc and copper ions had no effects on meat or carcass quality but bioavailability of trace minerals from algae appears to be greater than from inorganic salts	[342,355]
*Enteromorpha prolifera*	Feeding concentrated polysaccharide extracts improved growth performance, intestinal health, antioxidant status, and reduced *E. coli* population in cecum and diarrhea prevalence in weaned pigs	[328]
*Ulva* spp.	Nutrient digestibility in unprocessed biomass appears greater for swine than for poultry, with extracts of carbohydrates and protein from *U. lactuca* having the greatest nutritional value	[356]
*Ulva armoricana*	A sulfated polysaccharide-rich fraction (Ulvan) showed immunostimulatory responses *in vitro* using porcine intestinal epithelial cells; feeding an algal sulfated extract increased immunoglobulins in milk from sows	[333,335]
*Ulva lactuca*	A concentrated carbohydrate and protein fraction extract had greater nutritional value than unprocessed biomass, but reducing mineral content may be needed to optimize nutritional value; dietary inclusion up to 7% had no detrimental effects on growth performance, N balance, intestinal morphology, and increased some beneficial microbial populations and hemicellulose digestibility while decreasing DM and OM digestibility; feeding diets with 7% inclusion and a carbohydrase for 2 weeks improved meat tenderness, juiciness, overall acceptability, and docosahexaenoic acid and iodine content	[341,346,357,358]

**Table 10:** Summary of swine studies evaluating nutritional and biological responses from feeding red seaweed species.

Red Seaweed (*Rhodophyceae*)	Summary of Responses	References
*Chondrus crispus*	Has moderate OM digestibility but relatively low fermentability compared with other common seaweed species used in animal feed	[346]
*Gracilaria lemaneiformis*	Feeding diets containing 0.1% polysaccharide extract altered the gastrointestinal tract microflora and metabolites to improve antioxidant, anti-inflammatory, and immune responses, but had no effect on carcass characteristics and lean meat yield in finishing pigs	[300,338]
*Lithothamnium calcareum*	Digestible calcium source that increased number of piglets born alive per litter, milk yield and nutrient composition when fed to sows	[275,286,287]
*Palmaria palmata*	Had no effect on digesta characteristics and was fermented in the cecum compared with feeding algal fiber from *Eucheuma cottonii* and *Laminaria digitata*, *Palmaria palmata*	[346,359]
*Phymatolithon calcareum*	No effect on growth performance but improved antioxidant capacity in weaned pigs	[287]

**Table 11:** Summary of swine studies evaluating nutritional and biological responses from feeding seaweed blends and commercial products.

Blends and Commercial Products	Summary of Responses	References
Commercial seaweed extracts	Improved ADG and G:F, decreased fecal *E. coli*, increased fecal *Lactobacillus* sp., and enhanced immune function in weaned pigs	[334,360]
Commercial brown, green, and red seaweed blends	No consistent effect on sow and litter performance but increased beneficial fecal microbiota; diet inclusion of 0.5% did not provide comparable growth performance or reduce inflammatory responses compared with feeding pharmacological levels of ZnO; bioactive compounds in a blend of *Ascophyllum nodosum*, *Palmaria palmata*, and *Ulva lactuca* showed anti-inflammatory activity in porcine alveolar macrophages	[279,287,331]
Fermented rapeseed-seaweed blend	Feeding a blend of *Ascophyllum nodosum* (6%)*, Saccharina latissima* (6%)*, and rapeseed meal* (88%) to growing-finishing pigs tended to reduce parasitic *Ascaris suum* fecal egg count, reduced incidence of infection, and altered microbiome indicators of improved gut health; fermented rapeseed meal, but not *Ascophyllum nodosum* individually or in combination with *Saccharina latissima*, improved growth performance and intestinal development of weaned pigs comparable to feeding pharmacological levels of ZnO; reduced gut lymphocyte infiltration, improved colon mucosal barrier, and altered microbiota composition	[103,104,329,330]
Alginate oligosaccharides	Improved growth performance of weaned pigs by increasing antioxidant capacity, serum hormone concentrations, and intestinal digestive and absorption function; altered intestinal microflora composition and short chain FA concentrations to reduce intestinal inflammation and improve gut barrier integrity in weaned pigs; increased semen volume, sperm motility, increased beneficial sperm metabolites and proteins in mature boars	[151,285,296,297,302]
Laminarin and fucoidan extracts	Fed to sows in late gestation and lactation increased number of piglets born alive per litter, sow FI, milk yield, and growth rate of piglets; altered intestinal morphology and some intestinal microbiota independently but not in combination; improved ADG, G:F, and nutrient digestibility in weaned pigs; laminarin improved ADG and FI of weaned pigs by reducing pathogenic *Enterobacteriaceae* and increasing *Prevotellaceae*; feeding diets with fucoidan increased *Lactobacilli* spp. in cecum and butyric acid in cecum and colon, but laminarin and fucoidan increased fecal shedding of *Salmonella typhimurium*	[276,294,304,306,308]
Commercial *Palmaria palmata* powder	No effect on ADG, G:F, and fecal score of weaned pigs or gas emissions from manure	[361]
Seaweed powder	Maternal supplementation enhanced immune response of piglets	[261]
Prebiotic polysaccharides from *Laminaria digitata* and *L. hyperborea* + polyphenolic mixture from plant extracts	Feeding sows from late gestation through lactation had no effect on FI, litter birth weight, number of pigs born per litter, but reduced backfat losses during lactation and indicators of oxidative stress, and increased piglet growth rate, weaning weight, and total number of pigs born per litter at subsequent farrowing	[274]
Konjac glucomannan and ҡ-carrageenan	Improved sow performance and gut barrier function in offspring	[264]
Commercial *Ulva* + vitamins A and D	Dietary seaweed supplementation before vaccination enhanced humoral responses to Porcine Reproductive and Respiratory Syndrome virus vaccine	[336]
Commercial purified fucoidan drench derived from *Fucus vesiculosis*	No effect on growth performance, immune response, and intestinal morphology in weaned pigs	[332]
*Lithothamnion glaciale* and *Ascophyllum nodosum* extract	Dose dependent synergistic effects when fed in combination that improved gut barrier function and reduced inflammation using an ex vivo model of porcine ileal tissue challenged with *Salmonella enterica enteriditis*	[362]

**Table 12:** Summary of broiler studies evaluating nutritional and biological responses from feeding various seaweed species.

Seaweed Species	Summary of Responses	References
Brown (*Phaeophyceae*)		
*Ascophyllum nodosum*	Diet inclusion rate of 2% reduced heat stress and improved growth performance; adding 0.5 mg/tonne of extract reduced adverse physiological effects of prolonged heat stress exposure but had no effects on ADG and G:F; 2% seaweed and extract improved the abundance of beneficial microbes and improved morphology of the small intestine in heat stressed birds; supplementation reduced growth performance, cecal bacterial load and *Campylobacter* counts, and increased gene expression associated with gut integrity; diet inclusion up to 2% had no effect on growth performance, meat FA profile and lipid oxidation	[139,377,378,379,380]
*Hizikia fusiformis*	0.5% diet inclusion of fermented and non-fermented seaweed improved ADG and reduced FI and mortality rate	[381]
*Laminaria digitata*	Diet inclusion of 15% with and without carbohydrases impaired growth performance but enhanced meat quality by increasing pigment antioxidants, n-3 PUFA and iodine content; washing, ensiling, and extraction processes reduced nutritional value which made it unsuitable for use in poultry diets	[382,383]
*Saccharina latissima*	Washing, ensiling, and extraction processes reduced nutritional value which made it unsuitable for use in poultry diets	[383]
*Sargassum dentifebium*	Feeding finisher diets containing up to 6% raw or thermally treated (boiled and autoclaved) with and without enzyme supplementation for 39 days decreased ADG and final body weight, increased FI, but had no effect on carcass characteristics.	[384]
*Sargassum polycystum*	Dietary inclusion up to 5% improved pellet quality and growth performance with no effects on carcass yield and characteristics	[364]
*Sargassum wightii*	Diet inclusion up to 4% improved ADG, G:F, FI, carcass dressing percentage, proportion of leg, thigh, and breast weight, and meat quality	[385]
*Sargassum* spp.	Feeding up to 10% had no effect on growth performance but improved FA composition and antioxidant status of meat; 2% inclusion rate tended to improve ADG and G:F resulting from increased FI and nutrient digestibility	[375,386]
*Undaria pinnatifida*	Dietary inclusion of 0.5% fermented and non-fermented seaweed improved ADG and reduced mortality rate	[381]
Green (*Chlorophyceae*)		
*Enteromorpha prolifera*	Dietary inclusion resulted in upregulated detoxification enzyme pathway and regulated redox and mitochondrial apoptosis signaling pathways to alleviate liver and bursa damage caused by diets containing aflatoxin B_1_; altered gene expression to improve breast muscle yield	[387,388,389]
*Ulva lactuca*	Replacing 3% corn with seaweed had no effect on growth performance and nutrient retention, increased carcass dressing percentage and breast muscle yield, reduced serum lipid, cholesterol, and uric acid concentrations, and had no effect on serum enzymes and electrolytes; diet inclusion of 15% with or without supplemental carbohydrases had no effect on growth performance, improved the nutritional composition of meat, but reduced overall acceptability; washing, ensiling, and extraction processes reduced nutritional value and made it unsuitable for use in poultry diets	[382,383,390]
*Ulva laetevirens*	Diet inclusion of 5% or 10% with and without proteolytic enzyme treatment improved ADG and G:F, and feeding untreated seaweed reduced inflammation response	[391]
*Ulva rigida*	Diet inclusion up to 6% had no effect on ADG and carcass dressing percentage but improved intestinal morphology and reduced serum cholesterol and triglyceride concentrations	[392]
*Ulva* spp.	Pre-treatment of seaweed with protease and fibrolytic enzymes had no effect on growth performance, carcass traits, physiological measures, and meat quality; diet inclusion up to 3.5% had no linear or quadratic effects on growth performance, carcass, or meat quality traits but provide some improvement in meat shelf-life stability	[393,394]
Red (*Rhodophyceae*)		
*Chrondracanthus chamissoi*	Diet inclusion of 1.5% improved growth performance, intestinal morphology, and increased abundance of beneficial intestinal bacteria	[395]
*Chondrus crispus*	Supplementing diets with 0.30% to 0.40% improved growth performance, carcass and breast meat yield, decreased abdominal fat, increased bursa and thymus weight, and maintained intestinal pH	[396]
*Eucheuma cottonii*	Diet inclusion rate of 5% optimized growth performance but had no effect on carcass composition	[397]
*Eucheuma denticulatum*	Diet inclusion of 1 g/kg extract improved ADG and G:F, enhanced intestinal immunity and growth hormone receptor gene expression, decreased intestinal pathogens	[398]
*Gracilaria* spp.	Feeding up to 10% had no effect on growth performance but improved FA composition and antioxidant status of meat; feeding up to 2000 mg/kg concentrated polysaccharides improved ADG and G:F by altering antioxidant capacity, immune function, and intestinal microbiome composition; feeding up to 1% of diet had no effect on growth performance and meat quality, reduced oxidative stress and improved gut health; inclusion rate of 2.5% optimized growth performance but had no effect on carcass composition	[375,386,397,399]
*Kappaphycus alvarezii*	Feeding diets containing 1 g/kg seaweed extract improved growth performance and immune response by affecting intestinal gene expression of gut permeability and mucosal immunity; dietary inclusion up to 5% improved pellet quality and growth performance with no effects on carcass yield and characteristics	[364,400]
*Halymenia palmata*	Supplementing diets with up to 0.25% seaweed tended to linearly improve growth performance, increased total tract DM digestibility and fecal *Lactobacilli* sp., and decreased *Escherichia coli* and *Salmonella* sp. counts, improved intestinal morphology, decreased excreta ammonia and hydrogen sulfide emissions, and improved meat quality traits	[401]
*Lithothamnium calcareum*	Supplementing diets with 0.2% alleviated aflatoxin B_1_ liver damage	[402]
*Solieria chordalis*	Diet inclusion of 5% or 10% with and without proteolytic enzyme treatment had no effect on growth performance, reduced pre-cecal digestibility, and enzyme treatment did not improve health related measures	[391]
Blends and commercial products		
Brown, green, and red seaweed blend	Optimal inclusion rate was 0.5 to 1.0% for improved broiler growth performance and breast yield but higher doses enhanced prebiotic effects	[403]
*Laminaria japonica* powder and antibacterial peptide cecropin	Improved broiler growth performance, increased serum Newcastle disease antibody titers and lymphocytes, reduced *E. coli* and increased *Lactobacillus* in cecum	[404]
Laminarin and laminarin + fucoidan extracts from *Laminaria digitata*	Both extracts improved growth performance, intestinal morphology, and immune response but had no effect on *Campylobacter jejuni* proliferation	[405]
Laminarin extract from *Laminaria digitata*	Supplementation had no effect on growth performance and gut health	[406]
Fermented sea mustard by-product	Inclusion of 0.2% fermented sea mustard by-product improved growth performance, decreased *E. coli*, and increased *Lactobacillus* in excreta	[407]
Fermented kelp	Inclusion of 0.5% fermented kelp increased oxidative stability of meat and reduced ammonia emissions without affecting broiler growth performance	[408]
Sodium alginate oligosaccharides	Broilers challenged with *Salmonella* enterica had less body weight loss and mortality, reduced *Salmonella* colonization, improved intestinal barrier function and anti-inflammatory effects	[409]
Fucoxanthin derived from *Laminaria japonica*	Feeding 100 to 200 mg/kg provided an antimicrobial effect on *Staphylococcus* spp. in breast meat at 5 to 6 days of storage, improved antioxidant status, and had variable effects on drumstick color with no effect of growth performance	[410]
Polymannuronate derived from brown algae	Inclusion up to 0.4% improved growth performance, immune status, and antioxidant capacity	[411]
*Ascophyllum nodosum* extract	Addition of up to 5000 mg/kg extract to diets reduced fat accumulation in adipose tissue and liver by altering lipogenesis	[412]
Phlorotannin extract from *Ascophyllum nodosum*	Bactericidal activity was found for several *Campylobacter* spp. strains *in vitro* but no effect on reducing *Campylobacter* colonization *in vivo* or on growth performance	[413]
Microencapsulated blend of essential oils and *Ascophyllum nodosum* meal	Diet inclusion of 100 mg/kg improved growth performance, intestinal morphology, digestive enzyme activity, liver function, and metabolic biomarkers	[414]
Extracts from *Chondrus crispus* and *Sarcodiotheca gaudichaudii*	Extracts used in combination of tetracycline and streptomycin exhibited antimicrobial activity against *Salmonella* Enteritidis	[365]

**Table 13:** Summary of layer studies evaluating nutritional and biological responses from feeding various seaweed species.

Seaweed Species	Summary of Responses	References
Brown (*Phaeophyceae*)		
*Fucus serratus*/*F. vesiculosus*	Diet inclusion of 15% increased yolk carotenoids	[428]
*Hizikia fusiforme*	Diet inclusion of 0.5% improved egg production without affecting eggshell characteristics but feeding fermented seaweed had no effect on laying performance	[429]
*Sargassum* spp.	Feeding up to 3% inclusion rate improved egg production rate, egg mass, and IgM antibody titers with no effect on egg quality; diet inclusion up to 8% reduced egg production and yolk cholesterol content, had no effect on FI, egg weight, and feed conversion, albumen height, Haugh unit, but improved yolk color	[430,431]
*Sargassum binderi*	Diet inclusion of 16% improved lipid metabolism and egg quality by reducing cholesterol and low-density lipoprotein in plasma and egg yolk	[432]
*Sargassum dentifebium*	Processing method (sun-dried, boiled, autoclaved) and diet inclusion rate (3 and 6%) had variable effects on egg weight, yolk color, Haugh unit, and shell thickness	[433,434]
*Undaria pinnatifida*	Diet inclusion of 0.5% improved egg production without affecting eggshell characteristics but feeding fermented seaweed has no effect on laying performance	[429]
Green (*Chlorophyceae*)		
*Caulerpa lentillifera*	Diet inclusion of 1.5% increased egg production, antioxidant activity in egg yolk, and maintained liver health	[435]
*Cladophora* spp.	Diet addition improved egg weight, eggshell thickness, and hen body weight	[436]
*Enteromorpha prolifera*	Diet addition improved egg weight, eggshell thickness and hen body weight; diet inclusion of 1% to 3% had no effect on FI, egg laying rate, but improved feed conversion, egg weight, shell thickness, yolk color, immune function, and fecal microbial composition while reducing yolk cholesterol content	[436,437]
*Ulva* spp.	Ulvan extract added up to 1% of layer diets improved egg production, egg weight, feed conversion, eggshell strength, and yolk color while decreasing cholesterol in yolk and having some beneficial effects on improving antioxidant capacity in serum	[438]
Red (*Rhodophyceae*)		
*Chondrus crispus*	Diet inclusion up to 2% had no effect on FI, egg production, and body weight, but improved feed conversion, egg quality, and gut health; diet inclusion of 3% under short-term heat stress conditions reduced FI, ADG, G:F, but had minimal effects on performance in the long-term; diet inclusion up to 4% reduced *Salmonella Enteritidis* in ceca, maintained body weight and egg production, increased serum IgA and beneficial bacteria in ceca	[439,440,441]
*Kappaphycus alvarezii*	Adding up to 1.5% in pullet diets improved sexual maturity, egg production, egg weight, shell thickness, some internal egg qualities, cell-mediated and humoral immunity, and reduced lipid oxidation and cholesterol content of eggs	[415,416]
*Sarcadiotheca gaudichaudii*	Diet inclusion up to 2% had no effect on FI, egg production, body weight, but improved feed conversion, egg quality, and gut health; up to 4% diet supplementation reduced *Salmonella Enteritidis* in ceca, maintained body weight and egg production, increased serum IgA and beneficial bacteria in ceca	[439,440]
Blends and commercial products		
*Macrocystis pyrifera* + sardine oil	Diet inclusion of 10% with 2% sardine oil decreased egg weight and shell thickness and weight but improved yolk color, albumen height, Haugh unit, and EPA content in eggs	[431,442]
Post-extraction residue from *Macrocystis pyrifera*	Diet inclusion of 5% increased egg weight, shell thickness and weight, yolk color, albumen height, and Haugh unit	[443]
*Sargassum sincola* + sardine oil	Diet inclusion of 10% with 2% sardine oil decreased egg weight, shell thickness and weight, and yolk color but increased albumen height, Haugh unit, and EPA content in eggs	[431,442]
*Enteromorpha* spp. + sardine oil	Feeding diets containing 2% sardine oil and 10% *Enteromorpha* spp. increased DHA content in eggs	[442]
*Chondrus crispus and Sarcodiotheca gaudichaudii extracts*	Feeding purified extracts along with common antibiotics were effective in controlling *Salmonella* Enteritidis	[444]

**Table 14:** Ranking of food safety hazards in seaweed (adapted from [462]).

Hazard	Link to Food	Link to Feed	>2% of Total RASFF ^1^ Reports	Concern for ≥25% of Stakeholders	Hazard Score ^2^	Relative Hazard Severity ^3^
Arsenic	Possibly	Yes	Yes	Yes	1.67	Major
Cadmium	Possibly	Possibly	Yes	Yes	1.59	Major
Iodine	Yes	Yes	Yes	Yes	1.50	Major
*Salmonella*	Yes	Yes	No	Yes	1.50	Major
Lead	Possibly	Possibly	No	Yes	1.34	Moderate
Mercury	Possibly	Possibly	No	Yes	1.34	Moderate
Aluminum	Possibly	Possibly	Yes	No	1.34	Moderate
*Bacillus*	Yes	Limited data	No	Yes	1.33	Moderate
Norovirus	Yes	Limited data	No	Yes	1.33	Moderate
Dioxins and Polychlorinated biphenyls	Limited data	Limited data	No	Yes	1.17	Minor *
Brominated flame retardants	Limited data	Limited data	No	Yes	1.17	Minor *
Polycyclic aromatic hydrocarbons	Limited data	Limited data	No	Yes	1.17	Minor *
Other pathogenic bacteria	Possibly	Limited data	No	Yes	1.17	Minor *
Hepatits E virus	Limited data	Limited data	No	Yes	1.17	Minor *
Fluorine	Possibly	Possibly	No	No	1.09	Minor *
Pesticide residues	Limited data	Limited data	No	No	0.92	Minor *
Pharmaceuticals	Limited data	Limited data	No	No	0.92	Minor *
Marine biotoxins	Limited data	Limited data	No	No	0.92	Minor *
Allergens	Limited data	Limited data	No	Yes	0.92	Minor *
Micro- and nanoplastics	Limited data	Limited data	No	No	0.92	Minor *
Radionuclides	No	No	No	No	0.75	Minor

^1^ Rapid Alert System for Food and Feed (RASFF) is a system for reporting food safety issues within the European Union. ^2^ Hazard scores were assigned to each of four factors including occurrence in food, occurrence in feed, RASFF alerts, and survey responses from stakeholders in seaweed value chain indicating hazards considered to be of greatest concern. Final scores were aggregated and hazards were ranked by major (score 1.75 to 1.50), moderate (score 1.49 to 1.25), or minor (1.24 to 0.75). ^3^ Minor Relative Hazard and Severity score designated with “*” indicates data gaps for hazard safety assessment.

## Data Availability

No new data were created.

## Abbreviations

The following abbreviations are used in this manuscript:

3-NOP	3-nitrooxypropanol
ADF	acid detergent fiber
ADG	average daily gain
CP	crude protein
DCP	digestible crude protein
DDT	dichlorodiphenyltrichloroethane
DE	digestible energy
DHA	docosahexaenoic acid
DM	dry matter
DMI	dry matter intake
EE	ether extract
EPA	eicosapentaenoic acid
EU	European Union
FA	fatty acid
FI	feed intake
G:F	gain:feed
GHG	greenhouse gases
IgA	immunoglobulin A
IgG	immunoglobulin G
IMTA	integrated multi-trophic aquaculture
LCA	Life Cycle Assessment
LPS	lipopolysaccharide
MCR	methyl-coenzyme M reductase
MUFA	monounsaturated fatty acids
N	nitrogen
NDF	neutral detergent fiber
NFE	nitrogen free extract
OM	organic matter
PAH	polycyclic aromatic hydrocarbons
PBDE	polybrominated diphenyl ethers
PCB	polychlorinated biphenyls
PCDD	polychlorinated-dibenzo-p-dioxins
PCDF	polychlorinated dibenzofurans
PFAS	per- and polyfluoroalkyl substances
PUFA	polyunsaturated fatty acids
RASFF	Rapid Alert System for Food and Feed
SFA	saturated fatty acids
SWOT	Strengths, Weaknesses, Opportunities, and Threats
TDN	total digestible nutrients
TMR	total mixed ration
VFA	volatile fatty acid
ZnO	zinc oxide

## Appendix A

**Table A1:** Summary of *in vitro* studies evaluating the effectiveness of various brown seaweed species on reducing enteric methane emissions in ruminants.

Brown Seaweed Species	Reduced Emissions	No Effect on Emissions
*Agarum clathrum*	-	[505]
*Alaria esculenta*	-	[506,507,508]
*Alaria esculenta* extract	[509]	-
*Ascophyllum nodosum*	[508,510,511,512,513]	[505,511,514]
*Bifurcaria bifurcate*	-	[515]
*Chorda filum*	[512]	[505]
*Colpomenia peregrina*	-	[505]
*Colpomenia sinuosa*	[516]	-
*Colpomenia sp.*	-	[505]
*Cystoseira tamariscifolia*	-	[515]
*Cystoseira trinodis*	[516,517]	[210]
*Dictyopteris undulata*	-	[505]
*Dictyosiphon foeniculaceus*	-	[512]
*Dictyota bartayresii*	[516]	[210]
*Dictyoya* sp.	-	[505,517]
*Ecklonia arborea*	-	[505]
*Ecklonia maxima*	[511]	-
*Ecklonia radiata*	[518]	-
*Ecklonia stolonifera*	[519]	-
*Ectocarpus siliculosus*	-	[505,512]
*Egregia menziesii*	[520]	[505]
*Eisenia bicyclis*	[519]	-
*Elachista fucicola*	-	[512]
*Fucus distichus*	[520]	-
*Fucus evanescens*	-	[505]
*Fucus serratus*	-	[508,512]
*Fucus spiralis*	-	[505]
*Fucus vesiculosus*	[508,513,521]	[505,512,515]
*Halidrys siliquasa*	[512]	-
*Hedophyllum sessile*	-	[520]
*Himanthalia elongata*	-	[508,515]
*Hormophysa triquetra*	[516]	-
*Laminaria digitata*	[512]	[505,506,507,508,514]
*Laminaria farlowii*	-	[505]
*Laminaria japonica*	[511]	-
*Laminaria longicruris*	[521]	-
*Laminaria ochroleuca*	-	[517,522]
*Laminaria* sp.	-	[523]
*Lessonia nigrescens*	[511]	-
*Macrosystis pyrifera*	-	[505,520]
*Nereocystis luetkeana*	[520]	-
*Padina australis*	[516,523]	[210,517]
*Padina* sp.	[524]	-
*Pelvetiopsis limitata*	-	[505]
*Pelvetia canaliculata*	-	[506,507]
*Postelsia palmaeformis*	-	[520]
*Pylaiella littoralis*	[512]	-
*Saccharina groenlandica*	-	[520]
*Saccharina latissima*	-	[507,508,512,517,522,525]
*Saccharina latissima* extracts	[509]	-
*Sargassum filipendula*	-	[505]
*Sargassum fulvellum*	[519,526]	[511,527]
*Sargassum fusiforme*	[519,526]	-
*Sargassum horneri*	[528]	[505]
*Sargassum johnsonii*	-	[529]
*Sargassum mcclurei*	[530]	-
*Sargassum muticum*	[520,531]	[512]
*Sargassum* sp.	[524]	-
*Turbinaria ornata*	[524]	-
*Undaria pinnatifida*	[519,526]	-
*Zonaria farlowii*	[532]	-

**Table A2:** Summary of *in vitro* studies evaluating the effectiveness of various green seaweed species on reducing enteric methane emissions in ruminants.

Green Seaweed Species	Reduced Emissions	No Effect on Emissions
*Acrosiphonia* sp.	-	[506]
*Avrainvillea elliottii*	-	[505]
*Boergesenia forbesii*	-	[524]
*Caulerpa lentillifera*	-	[517]
*Caulerpa racemosa*	[524]	-
*Caulerpa taxifolia*	[516]	[210,517]
*Chaetomorpha linum*	[516]	[505]
*Cladophora coelothrix*	-	[517,533]
*Cladophora glomerata*	-	[512]
*Cladophora patentiramea*	[516]	[210,517]
*Cladophora rupestris*	-	[507]
*Cladophora sericea*	-	[505]
*Cladophora* sp.	[520]	-
*Derbesia tenuissima*	-	[516,517]
*Halimeda monile*	-	[505]
*Oedogonium* sp.	[516]	-
*Phyllospadix scouleri*	[520]	-
*Spongomorpha aeruginosa*	-	[505]
*Udotea flabellum*	-	[505]
*Udotea wilsonii*	-	[505]
*Ulva californica*	[520]	-
*Ulva fenestrata*	-	[512]
*Ulva intestinalis*	-	[505,515]
*Ulva lactuca*	-	[508]
*Ulva ohnoi*	[516]	[227,505,517]
*Ulva rigida*	-	[525]
*Ulva* sp.	-	[505,517,518]

**Table A3:** Summary of *in vitro* studies evaluating the effectiveness of various red seaweed species on reducing enteric methane emissions in ruminants.

Red Seaweed Species	Reduced Emissions	No Effect on Emissions
*Ahnfeltiopsis linearis*	-	[505]
*Amphiroa anceps* extract	[534]	-
*Asparagopsis armata*	[535]	-
*Asparagopsis taxiformis*	[210,244,497,505,512,515,516,520,530,534,535,536,537,538,539,540,541,542,543,544]	-
*Bonnemaisonia hamifera*	[518]	[512]
*Botryoglossum farlowianum*	-	[505]
*Calliarthron tuberculosum*	[520]	-
*Callophyllis flabellulata*	[544]	-
*Callophyllis* sp.	[520]	-
*Ceramium rubrum*	-	[505]
*Ceramium tenuicorne*	[512]	
*Champia parvula*	-	[505]
*Chondracanthus corymbiferus*	-	[505]
*Chondracanthus exasperatus*	-	[505]
*Chondracanthus tenellus*	[534]	
*Chondrus crispus*	[508,521]	[505,512]
*Corallina officinalis*	[520]	[505]
*Delesseria sanguinea*	-	[512]
*Devaleraea ramentacea*	-	[505]
*Dilsea carnosa*	[512]	-
*Endocladia muricata*	[520]	[505]
*Eucheuma cottoni*	-	[523]
*Eucheuma isoforme*	-	[505]
*Eucheuma spinosum*	[524]	-
*Euptilota formisissima*	[518]	-
*Furcellaria lumbricalis*	-	[512]
*Furcellaria* sp.	[521]	-
*Galaxaura rugosa*	-	[505]
*Gelidium* sp.	[524]	-
*Gigartina* sp.	[522]	[517]
*Gracilaria cervicornis*	-	[505]
*Gracilaria coronopifolia*	[524]	-
*Gracilaria gigas*	[524]	-
*Gracilaria parvispora*	[534]	-
*Gracilaria vermiculophylla*	[530]	[525]
*Gracilaria verrucosa*	[524]	[544]
*Gracilaria* sp.	[520,523,545]	-
*Grateloupia elliptica*	[534]	-
*Halymenia floresii*	-	[516,517,532]
*Halymenia* sp.	[524]	-
*Hypnea pannosa*	[516]	[517]
*Laurencia filiformis*	[516]	-
*Laurencia* sp.	-	[505]
*Mastrocarpus papillatus*	[520]	[505,544]
*Mastocarpus stellatus*	-	[506,507]
*Mazzaella japonica*	-	[544]
*Mazzaella splendens*	[520]	[544]
*Neoporphyra perforata*	-	[505]
*Neorhodomela larix*	-	[505]
*Odonthalia floccosa*	[520]	[505]
*Opuntiella californica*	[520]	-
*Palmaria mollis*	-	[544]
*Palmaria palmata*	[524]	[505,506,507,508]
*Palmaria palmata* extracts	[509]	-
*Phyllophora pseudoceranoides*	-	[505]
*Pikea californica*	-	[505]
*Plocamium cirrhosum*	[518]	-
*Plocamium violaceum*	-	[505]
*Polysiphonia* sp.	-	[505]
*Porphyra umbilicalis*	-	[508,512]
*Porphya* sp.	-	[505,506,507]
*Prionitis lanceolata*	[520]	[505,544]
*Prionitis* sp.	[520]	-
*Rhodomela confervoides*	-	[512]
*Sarcodiotheca gaudichaudii*	-	[505]
*Solieria tenera*	-	[505]
*Spermothamnion repens*	-	[505]
*Trichogloeopsis pedicellata*	-	[505]
*Vertebrata fucoides*	-	[512]
*Vertebrata lanosa*	-	[505]
*Vidalia colensoi*	[518]	-

**Table A4:** Summary of *in vivo* studies evaluating the effectiveness of various seaweed species on reducing enteric methane emissions in ruminants.

Seaweed Species	Reduced Emissions	No Effect on Emissions
Brown (*Phaeophyceae*)		
*Ascophyllum nodosum*	[197]	[195,198,216,224,259]
*Fucus serratus*	-	[199]
*Fucus vesiculosus*	-	[216]
*Himanthalia elongata*	-	[224]
*Sargassum johnsonii*	[202]	-
*Saccharina latissima*	-	[198,199]
*Sargassum muticum*	-	[198]
Green (*Chlorophyceae*)		
No reports	-	-
Red (*Rhodophyceae*)		
*Asparagopsis armata*	[204,205,206,238,546]	-
*Asparagopsis taxiformis*	[208,209,210,212,213,216,227,228,229,239,241,255,256,540,541,547]	-
*Chondrus crispus*	[214]	[199]
*Mazzaella japonica*	-	[544]
*Porphya* sp.	-	[257]
